# Dengue virus infections among European travellers, 2015 to 2019

**DOI:** 10.2807/1560-7917.ES.2022.27.2.2001937

**Published:** 2022-01-13

**Authors:** Céline M Gossner, Nelly Fournet, Christina Frank, Beatriz Fernández-Martínez, Martina Del Manso, Joana Gomes Dias, Henriette de Valk

**Affiliations:** 1 European Centre for Disease Prevention and Control, Solna, Sweden; 2 Santé publique France, Saint-Maurice, France; 3 Department for Infectious Disease Epidemiology, Robert Koch-Institut, Berlin, Germany; 4 Centro Nacional de Epidemiología & Spanish Consortium for Research in Epidemiology and Public Health, Instituto de Salud Carlos III, Madrid, Spain; 5 Istituto Superiore di Sanita, Rome, Italy

**Keywords:** dengue, travellers, Europe, surveillance, outbreak, vector-borne disease, travel

## Abstract

**Background:**

Dengue is a disease with major impacts on public health in tropical and subtropical countries. In Europe, in the past decade, few autochthonous outbreaks were described.

**Aim:**

We aimed to identify factors associated with frequency of dengue virus infection among European travellers and at assessing how surveillance data could support preparedness against autochthonous outbreaks within Europe.

**Methods:**

We performed a descriptive analysis of travel-related dengue cases reported by European countries from 2015 through 2019. Using flight passenger data, we calculated travellers’ infection rates (TIR). We investigated the following associations: (i) between TIR and incidence rate in selected countries of infection and (ii) between number of travel-related cases and occurrence of autochthonous outbreaks within Europe.

**Results:**

There were 11,478 travel-related dengue cases and the TIR was 2.8 cases per 100,000 travellers. Most cases were infected in Asia (71%), predominantly in south-eastern Asia. The TIR was highest among travellers returning from Asia (6.1/100,000). There was an association between the incidence rate in the country of infection and the TIR but no association between the number of travel-related cases and occurrence of autochthonous outbreaks in Europe.

**Conclusions:**

The likelihood of infection in travellers is a function of the ongoing epidemiological situation in the country of exposure. The number of travel-related cases alone is not sufficient to estimate the likelihood of autochthonous outbreaks where vectors are present in Europe. Additional contributing factors such as adequate vectorial capacity and suitable environmental conditions are required.

## Introduction

Dengue is an *Aedes*-borne disease affecting primarily people in the tropics and subtropics. It was estimated for 2010 that 390 million people are infected every year worldwide, of whom a quarter developed symptoms [1]. Dengue is the most frequent vector-borne viral illness in travellers [2].

Until 1930, dengue was endemic in the southern part of the European continent; several outbreaks driven by *Aedes aegypti* occurred in Greece and Turkey in the late 1920s with more than 1 million people affected [3]. In the mid-1950s, *Ae. aegypti* disappeared from Europe, the reasons remain unclear [3]. *Aedes albopictus* was first reported in Europe in 1979 in Albania, and is now established in all southern European countries [4,5]. While *Ae. albopictus* is considered less competent for dengue virus transmission than *Ae. aegypti*, it has been the driver of large dengue outbreaks such as those in Réunion, a French overseas department, since 2017 [6-8].

Since 2010 and until December 2021, autochthonous outbreaks of dengue have been reported in four European countries (excluding the overseas countries and territories and the outermost regions): Croatia, France, Italy and Spain [9]; these outbreaks were the result of introduction of the virus by viraemic travellers arriving from dengue-endemic areas into areas of Europe where the vectorial capacity of *Ae. albopictus* at the time was sufficient to facilitate autochthonous transmission.

Dengue is a mandatorily notifiable disease at the European level and cases are reported annually to the European Centre for Disease Prevention and Control (ECDC). We analysed surveillance data of travel-related dengue cases reported by European countries between 1 January 2015 and 31 December 2019 with two aims: firstly, to identify factors associated with frequency of infection among travellers, which should provide travellers, travel medicine clinics and public health authorities with the relevant information to mitigate this risk of infection; secondly, to assess how surveillance data could support preparedness against and timely control of autochthonous outbreaks in Europe. In this article, Europe refers to the 27 European Union member states, plus Iceland, Norway, Liechtenstein, and the United Kingdom, excluding their overseas countries and territories and their outermost regions.

## Methods

### Travel-related cases

We used travel-related cases reported through The European Surveillance System (TESSy) of ECDC [10]; data were extracted on 6 October 2020. A travel-related case was defined as an individual with a dengue virus infection acquired in a country other than the country of diagnosis.

We included probable and confirmed travel-related dengue cases. A probable case was a patient with fever, detection of specific IgM antibodies against dengue virus in a single serum sample and returning from an area with ongoing virus transmission within 2 weeks before symptoms onset [11]. A confirmed case was a patient meeting any of the following laboratory criteria: detection/isolation of the virus, viral nucleic acid or viral antigen from a clinical specimen, or detection of specific IgM antibodies in a single serum sample plus confirmation by neutralisation, seroconversion or fourfold antibody titre increase of specific antibodies in paired serum samples [11].

For time-related analysis we used, in order of preference, the date of onset, the date of diagnostics or the date of notification. If none of these dates were available or if the date used for statistics was earlier than any of the dates mentioned above, we used the date used for statistics, which is the only mandatory date field in TESSy and refers to any date between the infection date and the reporting date.

To describe the geographical distribution of the travel-related cases within Europe, we used the place of notification and, when not available, the place of residence of the cases; both variables are reported at the third level of the Nomenclature of Territorial Units for Statistics (NUTS-3) [12].

### Travellers

We obtained monthly travellers’ data for the period 2015 to 2019 from the International Air Transport Association (IATA) that captures passenger volume on commercial flights [13]. We used data for inbound flight passengers (i.e. passengers arriving to Europe via direct or indirect flights); we assumed that for cases detected in Europe, the inbound flight took place at a date relatively close to the date of onset and consequently the date of infection. We assumed that infection occurred in the departure country.

### Cases in the local population of the countries of infection and population estimates in these countries

We obtained the yearly number of dengue cases among the local population of the countries of infection through publications, official reports and the World Health Organization website [14-23]. Population data were extracted from the World Bank and the French National Institute for Statistics [24,25].

### Mosquito vector distribution and population estimates in Europe

For each year, we obtained data on establishment of *Ae. albopictus* at the NUTS-3 level from the VectorNet database and the French Ministry of Health website [5,26].

We used yearly human population data in European countries provided by Eurostat [27].

### Country classification and grouping

**Figure 1:**
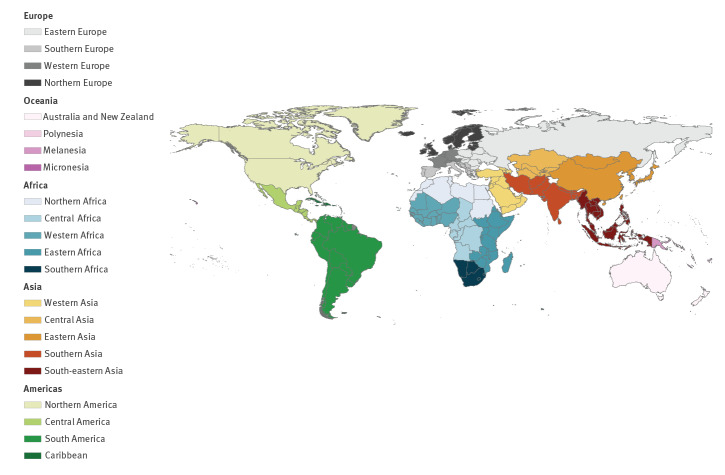
Regional grouping of countries following the United Nations Statistics Division

Because their epidemiological situation is distinct from mainland European countries, the European overseas countries and territories and outermost regions (e.g. Aruba, Cayman Islands, Madeira, Martinique, Réunion) were labelled as countries and their data analysed separately from mainland European countries’ data. We grouped countries by geographical region following the United Nations Statistics Division (Figure 1) [28].

Map produced on 22 October 2020. Administrative boundaries: EuroGeographics, Food and Agriculture Organization of the United Nations.

### Study inclusion criteria

The applied inclusion criteria aimed to account for possible errors in gathering or reporting of travel history/exposure of the cases and the lack of specificity of IgM serology testing [29,30]. Detailed information about the application of the inclusion criteria is provided in the Supplement (part A - Study inclusion criteria). We included: (i) probable and confirmed travel-related cases, (ii) European countries that submitted data every year and, over the whole period, provided country of infection for at least 50% of their cases (arbitrary cut off), (iii) cases with known country of infection (multiple countries of infection were recoded to unknown) and (iv) countries of infections associated with at least two cases, of whom one or more was a confirmed case, and that were either reported by two different reporting countries or reported over multiple years.

### Analysis

We first performed a descriptive analysis of case characteristics, focusing on place of residence, demographics, month of onset and country and region of infection. Considering the short incubation period of dengue (< 10 days), we considered that the month of onset was the same as the month of infection. As proxy for the risk of infection, we calculated the travellers’ infection rates per 100,000 travellers (TIR) and the 95% confidence intervals (CI) around the TIR estimates based on a Poisson distribution. The TIR was calculated following Formula 1: 


$$TIR=\frac{Number\;of\;travel-related\;cases\;infected\;in\;country\;(or\;region)\;A}{Number\;of\;travellers\;arriving\;to\;Europe\;from\;country\;(or\;region)\;A}*100,000$$

We identified seasonal patterns and trends in the number of travel-related cases and TIR by using centred moving averages: 3- and 6-month moving average to describe the seasonality (bi-annual and annual peaks, respectively) and 12-month moving average for the overall trend.

For the trend analysis, we used a harmonic regression model including Fourier terms for capturing seasonality. In this model, we adjusted for seasonality using three pairs of sine and cosine with 12, 6 and 3 months as length of the periods to capture both the two yearly peaks and to allow to capture the 'wavy' pattern in the data. This analysis was performed by regions.

We analysed the association between TIR for a given year and a given country of infection and disease incidence rate in the local population using a linear regression. We selected the four countries of infection (Cuba, French Polynesia, Réunion and Thailand) with the highest number of cases in their region, Americas, Oceania, Africa and Asia, respectively. For each selected country, the disease incidence rate in the local population was obtained by dividing the number of cases in the local population by the population estimate (cases/100,000 population).

To define the risk of autochthonous transmission in Europe, we assessed the association between the number of travel-related cases in receptive areas and the number of autochthonous outbreaks that occurred in Europe from 2015 through 2019 [9].

A receptive area was defined as a NUTS-3 region where *Ae. albopictus* was established and at a time when vectorial capacity was assumed sufficient to facilitate local transmission, estimated to be between 1 July and 31 October. The period of sufficient vectorial capacity was defined based on the recorded occurrence of autochthonous vector-borne transmission of dengue virus in Europe since 2010 (all outbreaks occurred during the period July to October) [9]. Per year and per European country, we selected the number of travel-related cases with date of onset between 1 July and 31 October and notified or residing in a NUTS-3 region where *Ae. albopictus* was established.

Alternatively, when the place of notification and place of residence were not available or not available in the right format (e.g. NUTS-2), we assumed that the geographical distribution of the travel-related cases was following the geographical population distribution of the country. Consequently, to estimate the number of cases in receptive areas (µ), we used the following Formula 2:


$$µ=N\;of\;cases\;from\;1\;Jul\;to\;31\;Oct*\;\frac{Pop\;\;in\;regions\;with\;established\;Ae.\;albopictus}{Total\;pop\;\;in\;the\;country}*100$$

where N is the number and pop the population.

We used Stata software release 14 (StataCorp. LP, College Station, United States) for all data management and statistical analyses.

### Ethical statement

The ECDC is an agency of the European Union established under Regulation 851/2004 and it acts under such legal framework. Within the field of its mission, the ECDC shall search for, collect, collate, evaluate and disseminate relevant scientific and technical data. This study included anonymised surveillance data of dengue cases, which have been collected through The European Surveillance System of ECDC. The use of such data does not require an ethical approval.

## Results

### General results

**Figure 2:**
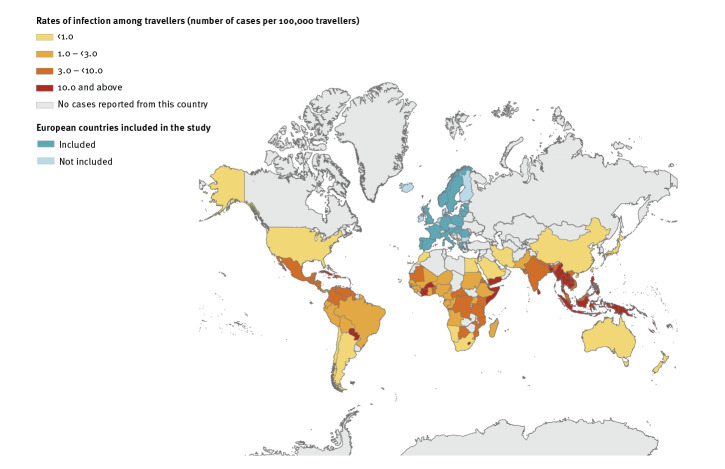
Rates of dengue virus infection among European travellers, per country of infection, and European countries included in the study, 2015–2019 (n = 11,478)

From 2015 through 2019, 11,478 travel-related dengue cases were reported in Europe, who had been were infected in 110 different countries around the world (Table 1; Figure 2). Nineteen European countries reported those cases (list provided in the Supplement, part A – Study inclusion criteria). The majority of the cases (91%) were confirmed cases (Table 2). Place of residence was available for 5,526 cases and among those, 99% (n = 5,457) were residing within Europe.

**Table 1:** Number of travel-related dengue cases reported in Europe and rates of infection, by country and region of infection, 2015–2019 (n = 11,478)

Region/country of infection	Number of travel-related cases	Rate of infection among travellers (cases/100,000 travellers)^a^	95% confidence interval^a^
**ASIA**	**8,144**	**6.1**	**6.0–6.3**
*South-eastern Asia*	*5,621*	*15.8*	*15.4–16.2*
Thailand	2,956	19.6	18.9–20.3
Indonesia	1,139	29.0	27.4–30.8
Philippines	445	12.6	11.4–13.8
Vietnam	331	10.0	8.9–11.1
Cambodia	278	45.7	40.5–51.5
Malaysia	226	7.3	6.4–8.3
Myanmar	131	31.7	26.5–37.6
Singapore	68	1.2	1.0–1.6
Laos	41	49.1	35.2–66.6
Brunei Darussalam	4	11.7	3.2–30.0
Timor-Leste	2	>100	NP^b^
*Southern Asia*	*2,479*	*8.1*	*7.8–8.4*
India	1,347	8.7	8.3–9.2
Sri Lanka	514	17.8	16.3.19.4
Maldives	368	18.1	16.3–20.1
Bangladesh	125	10.3	8.6–12.3
Pakistan	67	1.4	1.1–1.7
Nepal	55	6.3	4.8–8.3
Iran	3	0.1	< 0.1–0.3
*Eastern Asia*	*31*	*0.1*	*< 0.1–0.1*
China	25	0.1	< 0.1–0.1
Japan	6	< 0.1	< 0.1–0.1
*Western Asia*	*13*	*0.1*	*< 0.1–0.1*
Saudi Arabia	6	0.1	0.1–0.3
United Arab Emirates	5	< 0.1	< 0.1–0.1
Yemen	2	38.8	4.7–140.3
AMERICAS	2,079	1.2	1.1–1.2
*Caribbean*	*914*	*4.4*	*4.1–4.7*
Cuba	453	10.5	9.6–11.5
Dominican Republic	170	3.4	2.9–3.9
Jamaica	100	6.7	5.5–8.2
Guadeloupe, France	76	2.5	1.9–3.1
Martinique, France	38	1.4	1.0–2.0
Haiti	35	27.3	19.0–38.0
Antigua and Barbuda	14	3.2	1.8–5.4
Barbados	9	0.6	0.3–1.2
Dominica	4	9.9	2.7–25.4
Puerto Rico, United States	4	0.8	0.2–2.0
Bahamas	3	0.7	0.2–2.2
Leeward Antilles, the Netherlands	3	0.8	0.2–2.4
Trinidad and Tobago	3	1.0	0.2–3.0
Saint Lucia	2	0.5	0.1–1.7
*South America*	*654*	*2.2*	*2.0–2.4*
Brazil	299	2.5	2.2–2.8
Colombia	123	3.2	2.7–3.9
Paraguay	75	23.7	18.6–29.6
Ecuador	39	2.8	20.1–3.7
Venezuela	39	4.5	3.2–6.2
Peru	31	1.2	0.8–1.7
Argentina	18	0.3	0.2–0.5
French Guiana, France	14	1.7	0.9–2.8
Bolivia	9	1.9	0.9–3.5
Guyana	4	7.9	2.2–20.2
Chile	3	0.1	< 0.1–0.4
*Central America*	*503*	*3.9*	*3.6–4.3*
Mexico	303	3.2	2.9–3.6
Costa Rica	103	7.2	5.9–8.8
Guatemala	29	6.5	4.3–9.3
Nicaragua	22	8.6	5.4–13.1
Honduras	15	5.6	3.2–9.3
Panama	15	2.0	1.1–3.4
Belize	8	8.3	3.6–16.3
El Salvador	8	3.4	1.5–6.6
*Northern America*	*8*	*< 0.1*	*< 0.1– < 0.1* ^a^
United States (excluding Puerto Rico)	8	< 0.1	< 0.1– < 0.1^a^
**AFRICA**	**946**	**1.2**	**1.1–1.2**
*Eastern Africa*	*497*	*3.7*	*3.4–4.0*
Réunion, France	154	5.3	4.5–6.2
Seychelles	82	9.7	7.7–12.0
Kenya	76	3.5	2.7–4.3
Tanzania	66	5.0	3.9–6.4
Somalia	34	17.0	11.8–23.8
Ethiopia	15	2.0	1.1–3.3
Mozambique	11	3.1	1.6–5.6
Uganda	11	2.1	1.1–3.8
Mauritius	10	0.3	0.2–0.6
Comoros	9	11.7	5.4–22.3
Djibouti	9	7.2	3.3–13.6
Madagascar	8	1.0	0.5–2.1
Eritrea	6	4.6	1.7–10.1
Mayotte, France	6	3.3	1.2–7.1
*Western Africa*	*310*	*1.7*	*1.5–1.9*
Côte d’Ivoire	111	10.1	8.3–12.2
Burkina Faso	48	15.1	11.2–20.1
Nigeria	37	1.4	1.0–2.0
Senegal	23	0.9	0.6–1.4
Ghana	20	1.6	0.9–2.4
Togo	20	7.8	4.8–12.1
Benin	16	6.0	3.4–9.7
Mali	14	2.2	1.2–3.7
Mauritania	6	3.2	1.2–7.0
Guinea	4	1.8	0.5–4.5
Niger	4	2.4	0.7–6.2
Sierra Leone	3	2.2	0.4–6.3
Cabo Verde	2	0.1	< 0.1–0.3
Madeira, Portugal	2	< 0.1	< 0.1–0.1
*Central Africa*	*92*	*2.5*	*2.0–3.1*
Angola	28	1.9	1.3–2.8
Cameroon	25	2.7	1.8–4.0
Democratic Republic of the Congo	15	4.6	2.6–7.6
Congo (Brazzaville)	10	2.1	1.1–4.3
Equatorial Guinea	6	3.6	1.3–7.9
Gabon	5	1.6	0.5–3.7
Central African Republic	3	6.8	1.4–20.0
*Northern Africa*	*29*	*0.1*	*0.1–0.1* ^a^
Egypt	22	0.2	0.1–0.3
Sudan	5	2.1	0.7–4.8
Morocco	2	< 0.1	< 0.1– < 0.1^a^
*Southern Africa*	*18*	*0.2*	*0.1–0.4*
South Africa	7	0.1	< 0.1–0.2
Namibia	5	0.7	0.2–1.7
Botswana	3	3.7	0.8–10.7
Lesotho	3	>100	NP^b^
**OCEANIA**	**309**	**2.3**	**2.0–2.6**
*Polynesia*	*236*	*78.5*	*68.8–89.1*
French Polynesia, France	229	77.0	67.3–87.6
Tonga	5	>100	NP^b^
Samoa	2	99.3	12.0–358.5
*Melanesia*	*57*	*24.4*	*18.5–31.6*
New Caledonia, France	37	20.4	14.4–28.1
Fiji	12	29.8	15.4–52.0
Papua New Guinea	5	47.0	15.3–110.0
Vanuatu	3	>100	NP^b^
*Australia and New Zealand*	*14*	*0.1*	*0.1–0.2*
New Zealand and Cook Islands	8	0.4	0.2–0.8
Australia	6	0.1	< 0.1–0.1
*Micronesia*	*2*	*30.4*	*3.7–110.0*
Palau	2	30.4	3.7–110.0
**Global**	**11,478**	**2.8**	**2.8–2.9**

NP: not provided.

^a^ The similarity of value is due to the round-up of values.

^b^ Because of the extremely large TIR confidence interval, the exact TIR values for these infection rates are not shown but presented with an arbitrary cut-off of 100; these TIR should be considered with caution.

Map produced on 8 April 2020. Administrative boundaries: EuroGeographics, Food and Agriculture Organization of the United Nations.

**Table 2:** Characteristics of the travel-related dengue cases, Europe, 2015–2019 (n = 11,478)

	Number of travel-related cases	Percentage
Classification		
Probable	1,037	9%
Confirmed	10,441	91%
Sex		
Female	5,593	49%
Male	5,836	51%
Unknown	49	< 1%
Age group (years)		
0–4	56	< 1%
5–14	407	4%
15–24	1,727	15%
25–44	5,463	48%
45–64	3,132	27%
≥ 65	673	6%
Unknown	20	< 1%
Year of infection		
2015	1,892	17%
2016	2,403	21%
2017	1,738	15%
2018	1,833	16%
2019	3,612	31%
Month of infection		
January	870	8%
February	655	6%
March	948	8%
April	1,008	9%
May	916	8%
June	796	7%
July	967	8%
August	1,433	12%
September	1,120	10%
October	950	8%
November	1,127	10%
December	688	6%

**Figure 3:**
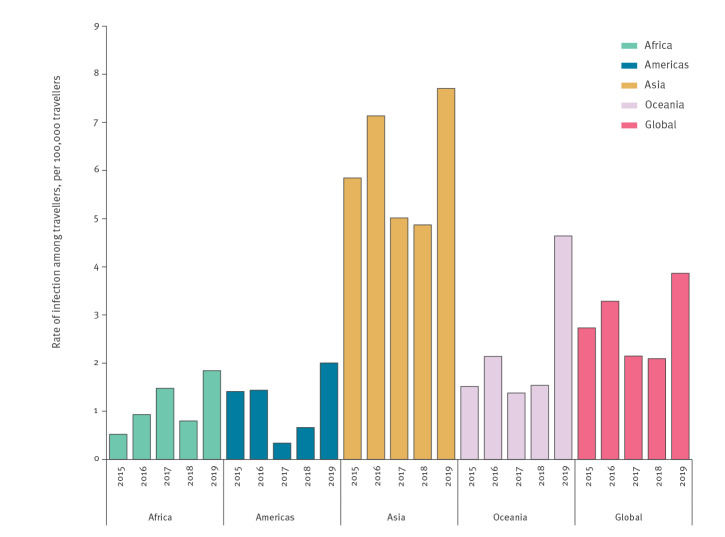
Rates of dengue virus infection among European travellers, by region of infection, globally and by year, Europe, 2015–2019 (n = 11,478)

The TIR over the period 2015 to 2019 was 2.8 cases per 100,000 travellers. The TIR was highest in 2019 (n = 3,612; TIR = 3.9) and lowest in 2018 (n= 1,833; TIR = 2.1) (Table 2; Figure 3). The median age of the cases was 35 years (interquartile range: 26–49), and men and women were almost equally affected (Table 2).

### Places of infection and likelihood of infection

From 2015 through 2019, cases primarily arrived from Asia (n = 8,144; 71%) (Table 1); of those cases, 69% and 30% were returning from south-eastern Asia and southern Asia. The TIR among travellers arriving from Asia was 6.1; for south-eastern Asia and southern Asia it was 15.8 and 8.1, respectively. There were peaks in the number of cases and TIR among travellers returning from south-eastern Asia in 2016 and 2019 (TIR = 20.6 and 19.7, respectively) and southern Asia in 2017 and 2019 (TIR = 9.3 and 10.3, respectively). For south-eastern Asia, cases arrived predominantly from Thailand (n = 2,956; TIR = 19.6) and Indonesia (n = 1,139; TIR = 29.0). For southern Asia, cases arrived predominantly from India (n = 1,347; TIR = 8.7) and Sri Lanka (n = 514; TIR = 17.8). Among Asian countries, the highest TIR were observed among travellers arriving from Timor-Leste (n = 2; TIR > 100), Laos (n = 41; TIR = 49.1) and Cambodia (n = 278; TIR = 45.7). 

Eighteen per cent of the cases were among travellers returning from the Americas (n = 2,079; TIR = 1.2). The Caribbean, South America and Central America accounted for 44%, 31% and 24% of these cases, respectively. There were peaks in the number of cases and TIR in relation to travel to the Caribbean and Central America in 2019 (TIR = 10.4 and 6.3, respectively). Most cases arrived from Cuba (n = 453; TIR = 10.5), Mexico (n = 303; TIR = 3.2) and Brazil (n = 299; TIR = 2.5). The highest TIR were among travellers arriving from Haiti (n = 35; TIR = 27.3) and Paraguay (n = 75; TIR = 23.7).

Eight per cent of the cases returned from Africa (n = 946; TIR = 1.2). Eastern Africa and western Africa accounted for 53% (TIR = 3.7) and 33% (TIR = 1.7) of these cases, respectively. Thirty-nine per cent of the cases from Africa were reported in 2019 (TIR = 1.8). From 2015 through 2019, the highest number of cases were related to Réunion (n = 154; TIR = 5.3) and Côte d’Ivoire (n = 111; TIR = 10.1) while the highest TIR were in relation to Lesotho (n = 3; TIR > 100) and Somalia (n = 34; TIR = 17.0).

Three per cent of the cases arrived from Oceania (n = 309; TIR = 2.3). The region Polynesia accounted for 76% of these cases. A peak in number of cases and TIR was observed among travellers returning from Oceania in 2019 (n = 137; TIR = 4.6). From 2015 through 2019, the majority of the cases were among travellers who arrived from French Polynesia (n = 229; TIR = 77.0) and New Caledonia (n = 37; TIR = 20.4). The highest TIR were among travellers arriving from Tonga (n = 5; TIR > 100) and Vanuatu (n = 3; TIR > 100).

### Seasonality of infections and trend analysis

Detailed results of the seasonality and trend analysis are provided in the Supplement (part B – Seasonality and trend analysis). Among travellers returning from south-eastern Asia, there were two seasonal peaks in cases and TIR, between March and May and in August. The first seasonal peak was mainly associated with travellers arriving from Thailand and Indonesia. The second seasonal peak was observed in travellers returning from most countries of the region. Among travellers retuning from southern Asia, the seasonal peak in cases and TIR was between August and November and was mostly attributed to India.

The seasonal peak in cases and TIR was between March and May for South America and between August and December for Central America and the Caribbean. The seasonal peaks in cases and TIR were in May and between August and November for travellers arriving from Polynesia.

There was a slight increasing trend in travel-related cases arriving from the Caribbean, southern Asia, and Polynesia with an average increase of 1–2% per month. In eastern Africa a 4% monthly increase in trend was observed.

### Association between disease incidence rate and travellers’ infection rate

We observed an association between the yearly disease incidence rate in the local population of countries of infection and the yearly TIR among travellers. The association appeared to be significant for three of the four selected countries, where an increase of 1 in the incidence rate in the country of infection was associated with a TIR increase of 1.44 for Cuba (95% CI: 0.51–2.37), 0.11 for French Polynesia (95% CI: 0.06–0.16) and 0.01 for Réunion (95% CI: 0.007–0.010). Detailed results are provided in the Supplement (part C – Linear regression analysis).

### Likelihood of autochthonous outbreaks within Europe

From 2015 through 2019, nine autochthonous vector-borne outbreaks of dengue occurred in Europe: six in France (one outbreak in 2015, three in 2018 and two in 2019) and three in Spain (two in 2018 and one in 2019) [9]. For four of the French outbreaks, investigations could point at a possible origin of the primary case: French Polynesia (2015 and 2018), Thailand (2019) and Cambodia (2019).

Among the European countries that reported travel-related cases, France, Germany, Greece, Italy, Malta, Romania, Slovenia and Spain had areas that could be receptive (Table 3). Place of notification and/or place of residence was available for the majority of the cases in all these countries, except for Italy and Malta (Table 3). For Italy and Malta, the estimated percentage of the population living in regions where *Ae. albopictus* is established grew from 81% to 100% in Italy from 2015 to 2019 and stayed at 93% in Malta.

**Table 3:** Estimated number of travel-related cases of dengue that could have led to autochthonous outbreaks in receptive areas and number of autochthonous outbreaks that actually occurred, per year and per country where *Aedes albopictus* is established, Europe, 2015–2019 (n = 11,478)

Year	France	Germany	Greece	Italy	Malta	Romania	Slovenia	Spain	Total
Number of travel-related cases between 1 July and 31 October									
2015	156	213	1	68	1	1	1	63	504
2016	177	239	0	45	1	3	3	92	560
2017	126	202	0	42	2	0	0	52	424
2018	146	186	0	51	0	1	0	93	477
2019	400	397	3	135	1	5	1	89	1,031
**2015–2019**	**1,005**	**1,237**	**4**	**341**	**5**	**10**	**5**	**389**	**2,996**
Number of travel-related cases between 1 July and 31 October, with place of notification or place of residence known									
2015	155	213	1	0	0	1	1	61	432
2016	177	239	0	0	0	3	3	91	513
2017	126	202	0	0	0	0	0	52	380
2018	146	186	0	0	0	1	0	91	424
2019	400	397	3	0	0	5	1	88	894
**2015–2019**	**1,004**	**1,237**	**4**	**0**	**0**	**10**	**5**	**383**	**2,643**
Percentage of population in regions where *Aedes albopictus* is established (%)									
2015	34	0	27	81	93	9	31	30	32
2016	36	0	76	99	93	9	57	33	39
2017	43	0	49	100	93	9	57	42	41
2018	55	1	83	100	93	17	57	42	45
2019	65	2	83	100	93	25	73	48	49
Number of cases notified or residing in receptive areas									
2015	113	0	0	55^a^	1^a^	0	0	48	217
2016	117	2	0	45^a^	1^a^	1	2	42	210
2017	91	2	0	42^a^	2^a^	0	0	45	182
2018	128	7	0	51^a^	0^a^	1	0	48	235
2019	373	14	3	135^a^	1^a^	3	1	44	574
**2015–2019**	**822**	**25**	**3**	**328** ^a^	**5** ^a^	**5**	**3**	**227**	**1,418**
Number of local transmission events that occurred									
2015	1	0	0	0	0	0	0	0	1
2016	0	0	0	0	0	0	0	0	0
2017	0	0	0	0	0	0	0	0	0
2018	3	0	0	0	0	0	0	2	5
2019	2	0	0	0	0	0	0	1	3
**2015–2019**	**6**	**0**	**0**	**0**	**0**	**0**	**0**	**3**	**9**

A receptive area was defined as a NUTS-3 region where *Aedes albopictus* was established and at a time when vector capacity was sufficient to facilitate local transmission, estimated to be between 1 July and 31 October.

^a^ Estimated based on Formula 2.

There were 1,418 travel-related cases in receptive areas from 2015 through 2019: of those, 822 were in France, 328 were in Italy and 227 were in Spain. In France, we estimated that the highest number of cases arriving in receptive areas was in 2019 with 373 cases, which coincided with the occurrence of two autochthonous outbreaks. In Spain, the number of cases in receptive areas remained relatively constant over time, ranging from 42 to 48 cases per year, while outbreaks only occurred in 2018 and 2019. In Italy, the number of cases in receptive areas over the study period was ca 44% higher than in Spain but no autochthonous outbreaks were reported.

## Discussion

We analysed more than 11,000 dengue cases reported from different European countries from 2015 through 2019. As we pooled together data from very diverse European countries, and because we captured a diverse group of people with different travel culture and behaviours, our results can be used to assess the risk of dengue virus infection among international travellers. Considering that the vast majority of the travel-related cases were residing within Europe, we could qualify the studied population as 'European travellers'.

As described in other studies, overall, travel-related dengue cases reported in Europe were primarily infected during a stay in Asia (mostly south-eastern and southern Asia), followed by the Americas, Africa and, finally, Oceania [31-33]. Overall, the estimated risk of infection for European travellers matches the known/described distribution of dengue worldwide. We were, however, surprised to observe that the risk of infection in Eastern Africa was similar to the risk of infection in Central America and the Caribbean. We therefore analysed further the data on travellers from Africa (not shown here). 

Variations in the distribution of regions of infection were observed among the European countries (data not shown); Spain had a comparatively large proportion of cases arriving from Central and Southern America, and France had a large proportion of cases arriving from Polynesia. Studies conducted on travellers arriving in other regions than Europe showed a different geographical distribution of cases compared with our study [34-36]. Those variations can easily be explained by differences in travel habits and preferences, which are deeply linked with historical and cultural connections between countries around the world.

International travellers are well known sentinels for disease surveillance [33,37,38]. They provide crucial information on disease occurrence in the visited countries, which is particularly relevant for countries with limited available surveillance data (e.g. because of limited laboratory capacity). For instance, we observed a striking increase in number of cases and TIR among travellers arriving from Côte d’Ivoire in 2017 and in 2019 (data not shown), years when outbreaks were reported in the country [39,40].

We highlighted that TIR provide more precise estimates of the risk of infection than case numbers, which are biased by the number of travellers. For instance, while Thailand was the country from where by far the largest number of cases arrived, the risk of infection was higher in less popular travel destinations such as Thailand’s neighbouring countries Laos, Myanmar and Cambodia. The relevance of the TIR should however be interpretated using the confidence intervals, which indicated that some of the highest TIR were likely to be artefacts due to low number of cases and travellers (i.e. for Lesotho, Timor-Leste, Tonga and Vanuatu). 

When data on travel-related cases are available, we consider that TIR are a better estimate of the likelihood of infection than the incidence rate in endemic countries. Firstly, the sensitivity of surveillance systems around the world is extremely variable, which makes comparison and therefore the assessment of the relative risk per country difficult. Secondly, considering the large proportion of individuals with immunity to at least one serotype of dengue virus in some dengue-endemic countries, the incidence rate in those countries does not reflect the actual level of virus circulation and therefore the risk for the European travellers, who can be considered as an immunologically naïve population [41,42]. This was exemplified by the difference in association observed between the incidence rates in Cuba, French Polynesia and Réunion and the respective TIR among travellers but also the lack of association between the incidence rate in Thailand, a country of very high endemicity, and the TIR among travellers returning from Thailand.

When visiting an area endemic for dengue, described factors for increased risk of infection include: timing of the visit when there is high vectorial capacity (e.g. following the rainy season), prolonged duration of the visit, conditions of travel (e.g. visiting friends and relatives, stay in accommodations with or without air-conditioning and/or window screens) and activities increasing exposure to mosquitoes (e.g. outdoor/active tourism) [43-45]. Most European countries are not reporting information on the length of stay, purpose of the travel and activities performed, and therefore we could not quantify the importance of these risk factors for European travellers. Also, we did not know where the cases were within the countries of infection. Considering the important diversity in climates among sub-national regions, we could not attempt to correlate the occurrence of travel-related cases with the period of higher vector capacity in countries of infection.

Traveller data from IATA do not include age and sex of the travellers; hence we could not define rates of infection among different age groups and sex. Also, traveller numbers based on air travel may be underestimated for destinations frequently reached via land borders or on locally bought separate air tickets, and overestimated for destinations with large airports with a high volume of international travellers. Calculated TIR would, respectively, be overestimated for harder-to-reach and somewhat underestimated for heavily travelled destinations.

We did not see a direct relationship between the number of travel-related cases in receptive areas and the likelihood of autochthonous outbreaks of dengue in Europe. In France, years with the highest number of cases in receptive areas (2018 and 2019) were indeed years when autochthonous outbreaks occurred and in Spain, one of the years with a higher number of cases also corresponded to a year with local transmission. However, the number of travel-related cases in receptive areas was not proportional to the number of autochthonous outbreaks occurring in a defined year, although it cannot be excluded that there were undetected clusters of autochthonous transmission. In addition, the number of travel-related cases in receptive areas was higher in Italy than in Spain, but no autochthonous outbreaks were detected in Italy until 2020 [46]. This emphasises that the number of travel-related cases arriving in receptive areas alone is not predictive of the occurrence of local outbreaks in these areas. It is the combination of several critical factors that triggers the start of a dengue outbreak in Europe; those factors include the force of introduction of the virus (i.e. number of viraemic travel-related cases), vector capacity, environmental conditions (e.g. temperature), compatibility of the viral strain with the local vector populations, level of interaction between humans and vectors, timeliness of the detection of the primary cases and timeliness and completeness of the vector control activities around the primary cases [47]. This finding is in line with the result of a similar analysis on the likelihood of chikungunya autochthonous outbreak in Europe [38].

When assessing the likelihood of autochthonous outbreaks of dengue from travel-related cases in receptive areas, we ignored three aspects: Firstly, not all cases are able to transmit the virus onward; it was estimated that ca 21% of the travel-related dengue cases are viraemic upon return to Europe [33]. Secondly, we considered that presence of an established local *Ae. albopictus* population was synonymous to sufficient vectorial capacity for autochthonous transmission. However, in France, local transmission was observed on average 6.5 years after the establishment of the competent vector [47]. Thirdly, approximately three quarters of the people infected with dengue virus may never be diagnosed because they remain asymptomatic and yet have the potential to transmit the virus onward [1,48]. While the first two aspects made us overestimate the number of cases that may trigger autochthonous outbreaks, the latter made us underestimate this same number of cases. For Italy and Malta, we had to estimate the number of cases in receptive areas, assuming that the distribution of cases was proportionate to the population. Considering the very large proportion of the population living in areas where *Ae. albopictus* is established in these two countries, we assessed that the estimated numbers should be relatively close to the actual number of cases detected in these areas.

Since the first autochthonous outbreak in mainland Europe in 2010 and until December 2021, 23 vector-borne autochthonous outbreaks of dengue have been recorded in mainland Europe, the largest of them included 11 cases in Italy in 2020 [9]. In comparison, there have been five vector-borne autochthonous outbreaks of chikungunya, two of which involved more than 300 cases and occurred 10 years apart in Italy (2007 and 2017) [49]. The comparatively higher number of introductions to Europe of dengue compared with chikungunya virus may explain that there were more autochthonous outbreaks of dengue than chikungunya outbreaks. Autochthonous dengue outbreaks stayed limited in size, even though some outbreaks were detected late, allowing transmission to continue for up to 3 months [47]. This indicates that European populations of *Ae. albopictus* may be better suited for chikungunya than for dengue virus transmission [47].

*Aedes albopictus* is progressively colonising new areas of Europe, increasing the number of areas at risk of autochthonous transmission [50,51]. *Aedes aegypti*, the main vector of dengue in most places around the world, is already established close to Europe (in Madeira, Portugal and on parts of the Black Sea shore) [52]. Its (re-)establishment in continental Europe would increase the risk of autochthonous outbreaks in Europe considerably.

Dengvaxia by Sanofi Pasteur (Paris, France) is the first licenced dengue vaccine [53,54]; this tetravalent chimeric yellow fever virus–dengue virus (CYD-TDV) vaccine targets people with previous exposure to dengue virus, hence requiring pre-vaccination serological screening. To date, the European Medicine Agency (EMA) has granted a marketing authorisation for this vaccine for individuals 9–45 years-old, living in an endemic area (e.g. some European overseas countries and territories and outermost regions) and who already had a prior dengue virus infection [55]. This excludes travellers, for whom the EMA does not recommend vaccination.

We made the choice not to include 2020 and 2021 data to avoid bias related to the coronavirus disease (COVID-19) pandemic. While the impact of the pandemic on the importation of dengue cases remains to be assessed, we can expect that owing to the travel restrictions applied in several countries in 2020-2021, the number of travellers will have dropped drastically and the proportion among different traveller types (e.g. tourism, business or visiting friends and relatives) will have been modified. It could also be possible that the proportion of undiagnosed cases was larger because of, among other things, reduced access to care and diagnostics in Europe. At this stage, we assume that the results presented in this study will be applicable after the COVID-19 pandemic. However, many questions remain, such as: Will the travel patterns be similar to before the pandemic? How severely have the vector control activities been affected worldwide? How will the global investments into molecular diagnostic capacity impact surveillance of dengue around the world? 

## Conclusion

Travellers should receive advice on how to prevent insect bites and should be reminded to seek prompt medical attention in case of febrile illness during their stay or upon their return to Europe. In addition, travellers returning to receptive areas should be advised to continue protecting themselves against mosquito bites after their arrival. The ECDC collects data on travel-related dengue cases on an annual basis and therefore, assessments on the risk of infection are provided retrospectively. Timely surveillance of ongoing outbreaks occurring globally are performed through epidemic intelligence activities and communicated on a monthly basis through the Communicable Disease Threat Reports. 

## Supplementary Data

394000 Supplement
