# Supplementary material for: Dengue virus infections among European travellers, 2015 to 2019
Source: Euro Surveill. 2022 Jan 13;27(2):2001937. doi: 10.2807/1560-7917.ES.2022.27.2.2001937 (PMC8759115; doi:10.2807/1560-7917.ES.2022.27.2.2001937)
Supplement: Supplement [file 20-01937_GOSSNER_Supplement.pdf]

Supplement

This supplementary material is hosted by *Eurosurveillance* as supporting information alongside the article *Dengue infections among travellers arriving to Europe, 2015-2019*, on behalf of the authors, who remain responsible for the accuracy and appropriateness of the content. The same standards for ethics, copyright, attributions and permissions as for the article apply. Supplements are not edited by *Eurosurveillance* and the journal is not responsible for the maintenance of any links or email addresses provided therein.

Part A – Study inclusion criteria

All cases of dengue reported through The European Surveillance System between 2015 and 2019
Number of reporting countries: 26 (AT, BE, DE, EE, EL, ES, FI, FR, HR, HU, IE, IS, IT, LT, LU, LV, MT, NL, NO, PL, PT, RO, SE, SI, SK and UK)
Number of cases reported: 13788
Number of countries of infection: 135

↓

Probable and confirmed travel-related cases
Number of reporting countries: 26 (AT, BE, DE, EE, EL, ES, FI, FR, HR, HU, IE, IS, IT, LT, LU, LV, MT, NL, NO, PL, PT, RO, SE, SI, SK and UK)
Number of cases reported: 13755
Number of countries of infection: 133

↓

Reporting countries that submitted data every year and provided country of infection for at least 50% of their cases over the study period
Number of reporting countries: 19 (AT, DE, EE, EL, ES, FR, HU, IT, LT, LV, MT, NO, PL, PT, RO, SE, SI, SK and UK)
Number of cases reported: 12759
Number of countries of infection: 133

↓

Cases with known country of infections
Number of reporting countries: 19 (AT, DE, EE, EL, ES, FR, HU, IT, LT, LV, MT, NO, PL, PT, RO, SE, SI, SK and UK)
Number of cases reported: 11503
Number of countries of infection: 133

↓

Countries of infections that were associated to at least two cases, of which one or more is a confirmed case, and that were either reported by two different reporting countries or reported over multiple years
Number of reporting countries: 19 (AT, DE, EE, EL, ES, FR, HU, IT, LT, LV, MT, NO, PL, PT, RO, SE, SI, SK and UK)
Number of cases reported: 11478
Number of countries of infection: 110

Note: AT (Austria), BE (Belgium), DE (Germany), EE (Estonia), EL (Greece), ES (Spain), FI (Finland), FR (France), HR (Croatia), HU (Hungary), IE (Ireland), IT (Italy), LT (Lithuania), LV (Latvia), MT (Malta), NL (The Netherlands), NO (Norway), PL (Poland), PT (Portugal), RO (Romania), SE (Sweden), SI (Slovenia), SK (Slovakia) and UK (The United Kingdom)

Part B – Seasonality and trend analysis

Number of travel-related cases of dengue among travellers arriving from **South-Eastern Asia** and rates of infection (TIR), by month, and 3-, 6- and 12-month moving averages, 2015-2019

Rate of infection

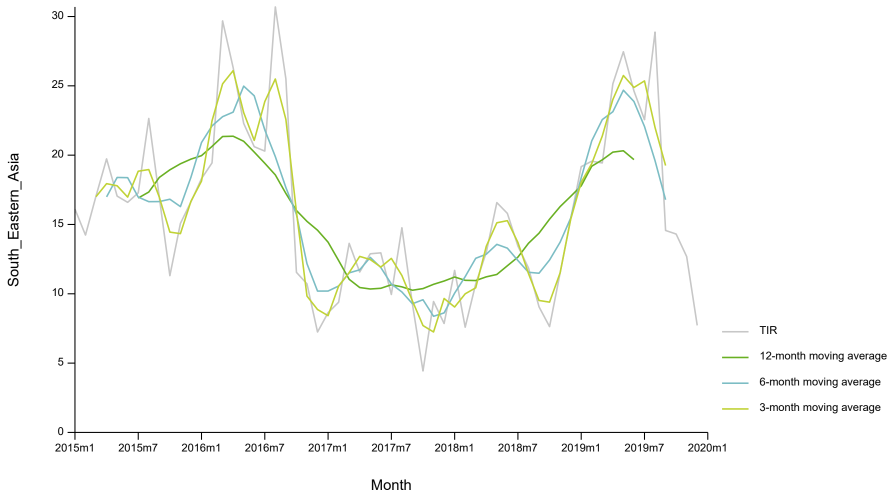

Number of cases

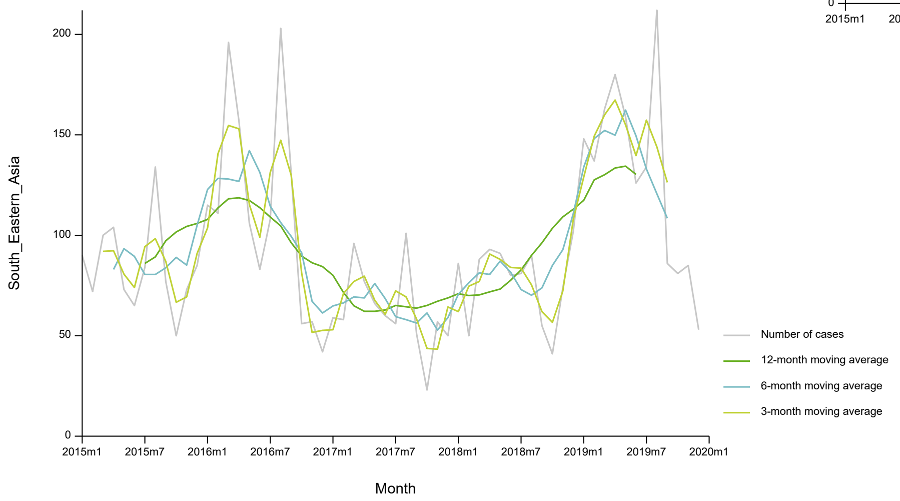

Trend adjusted for seasonality

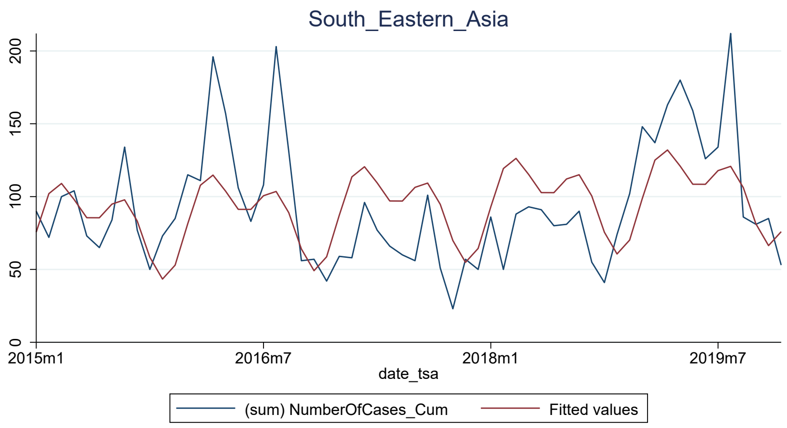

Number of travel-related cases of dengue among travellers arriving from **Southern Asia** and rates of infection (TIR), by month, and 3-, 6- and 12-month moving averages, 2015-2019

Rate of infection

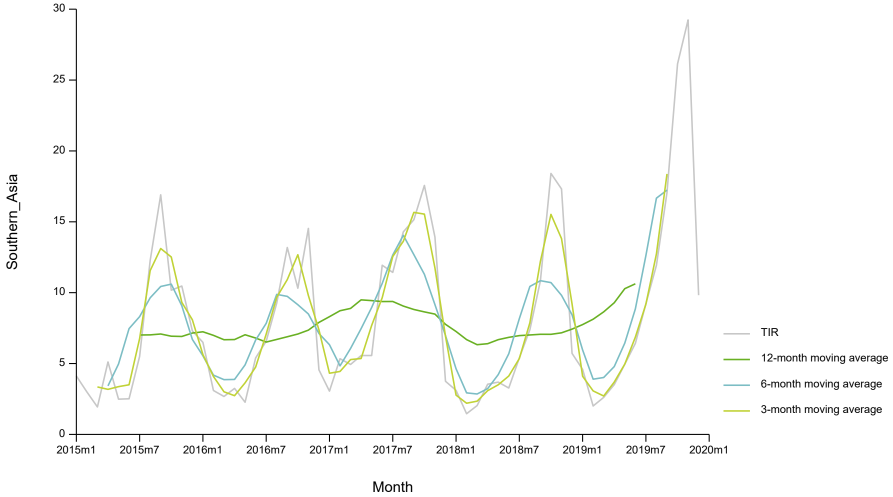

Number of cases

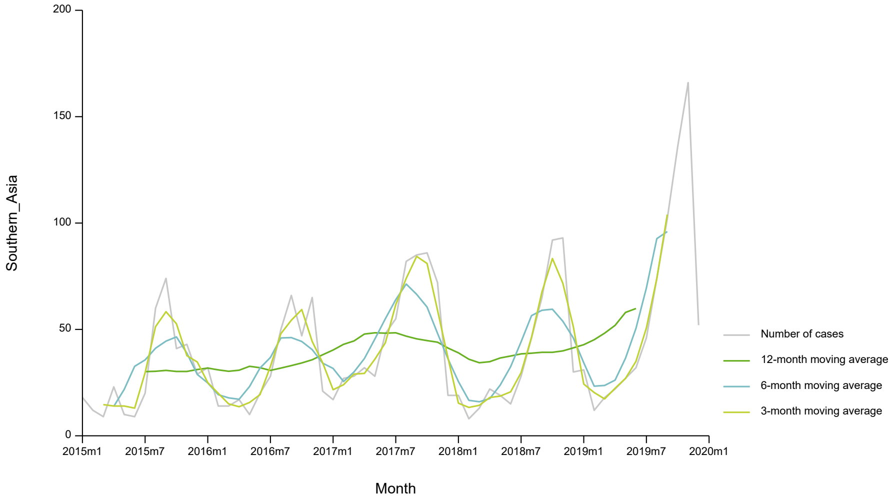

Trend adjusted for seasonality

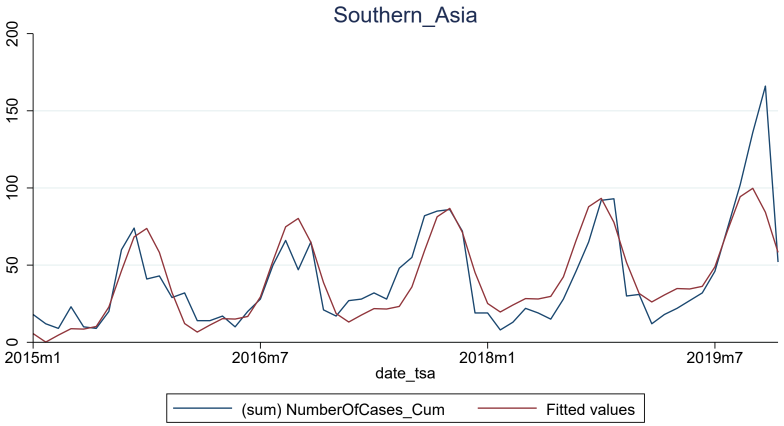

Number of travel-related cases of dengue among travellers arriving from the **Caribbean** and rates of infection (TIR), by month, and 3-, 6- and 12-month moving averages, 2015-2019

Rate of infection

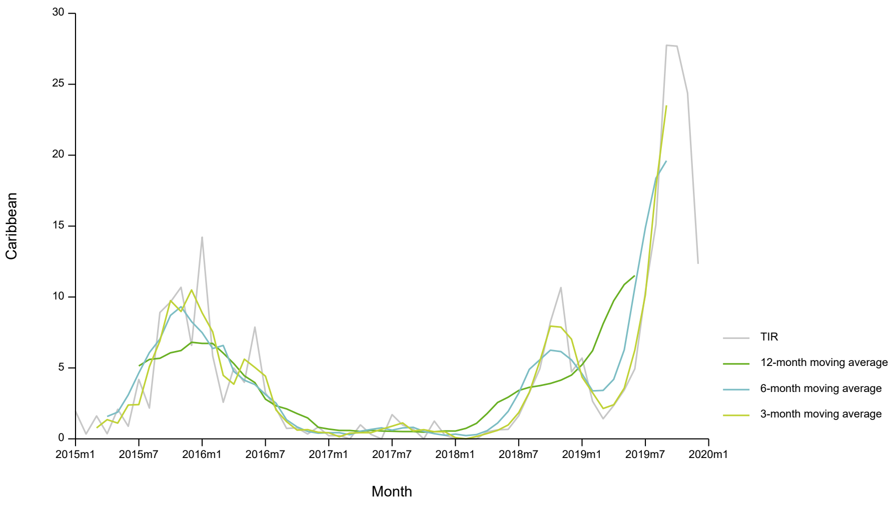

Number of cases

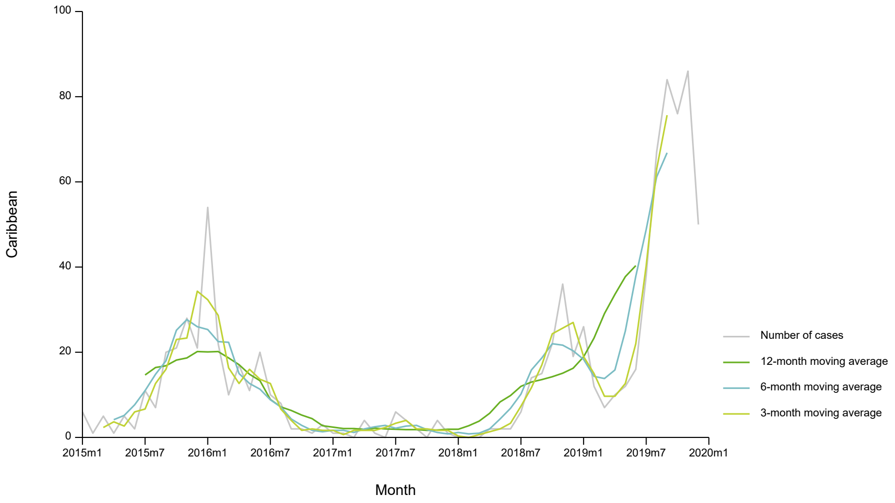

Trend adjusted for seasonality

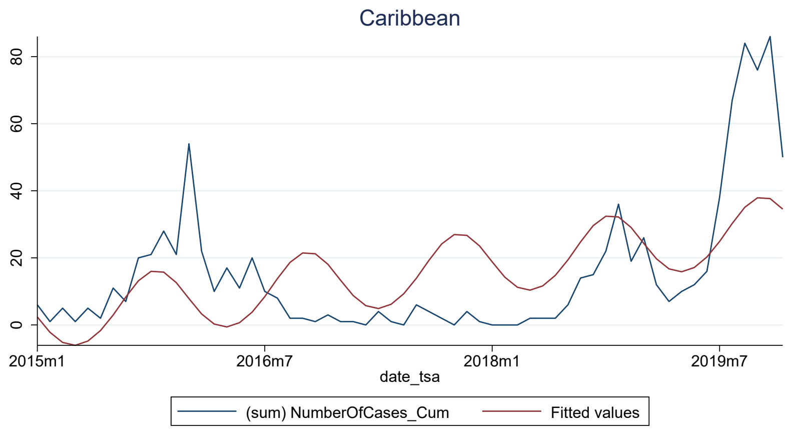

Number of travel-related cases of dengue among travellers arriving from **South America** and rates of infection (TIR), by month, and 6 and 12-month moving averages, 2015-2019

Rate of infection

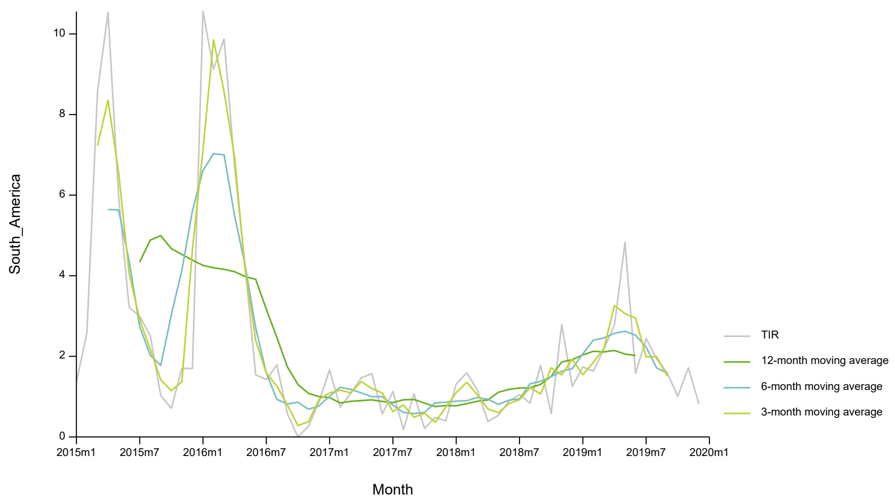

Number of cases

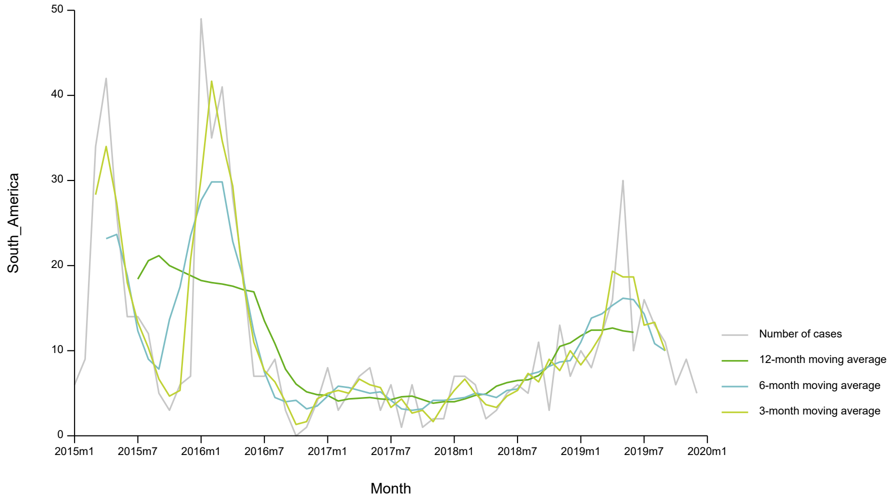

Trend adjusted for seasonality

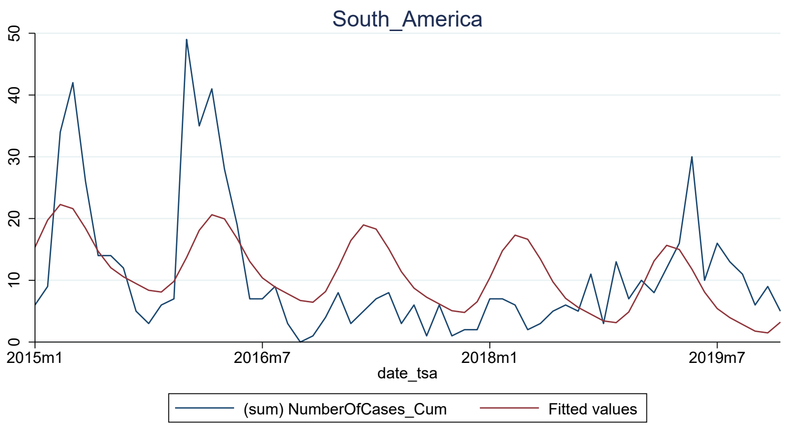

Number of travel-related cases of dengue among travellers arriving from **Central America** and rates of infection (TIR), by month, and 3-, 6- and 12-month moving averages, 2015-2019

Rate of infection

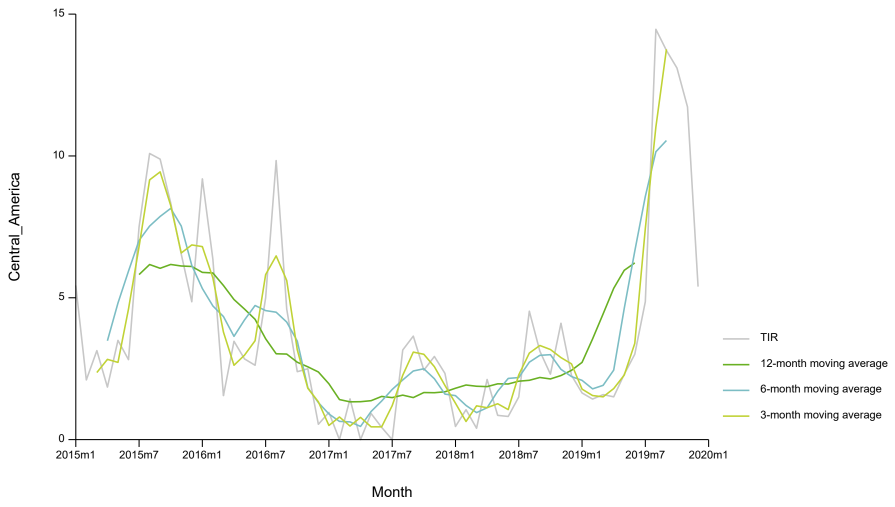

Number of cases

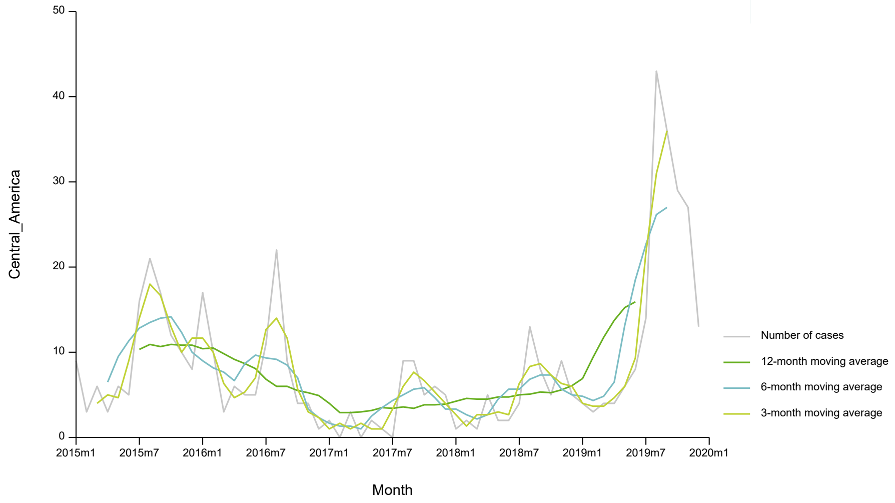

Trend adjusted for seasonality

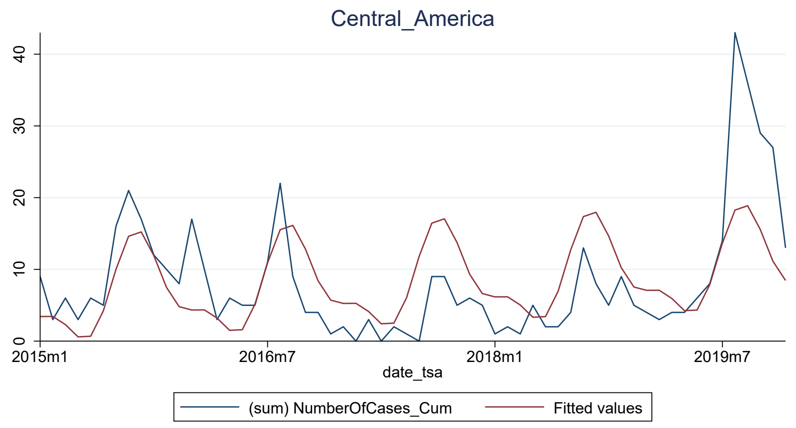

Number of travel-related cases of dengue among travellers arriving from **Eastern Africa** and rates of infection (TIR), by month, and 3-, 6- and 12-month moving averages, 2015-2019

Rate of infection

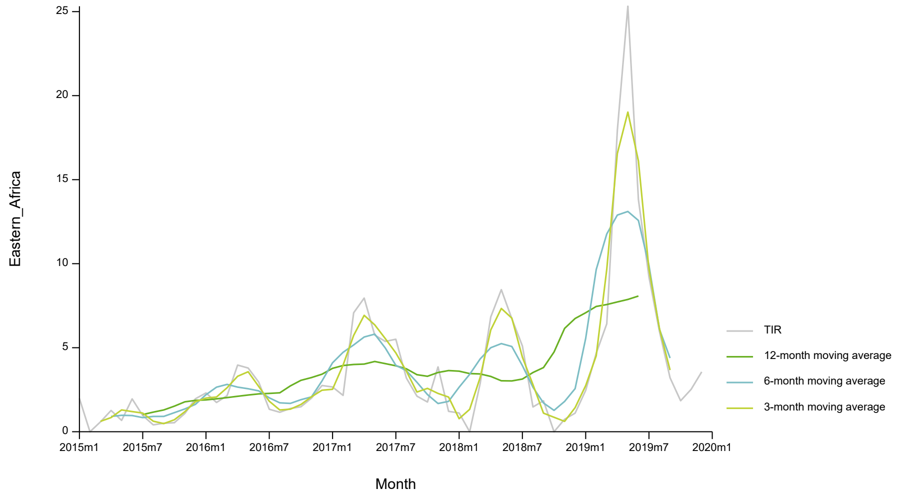

Number of cases

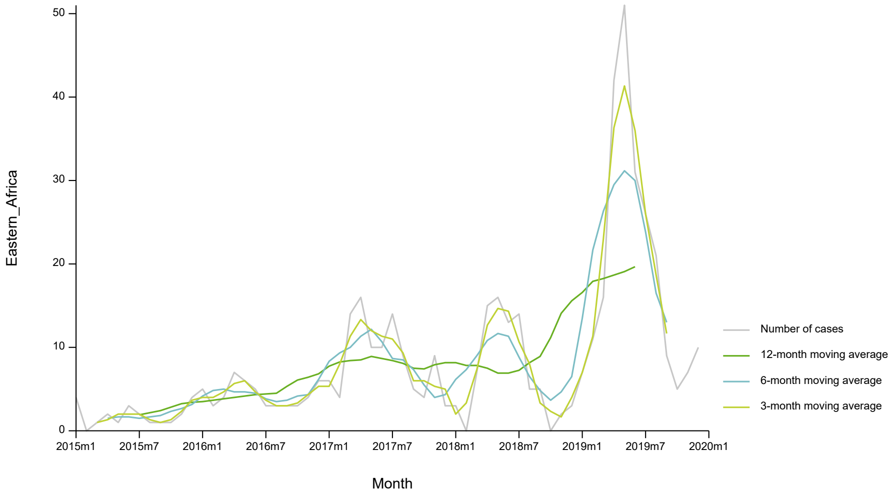

Trend adjusted for seasonality

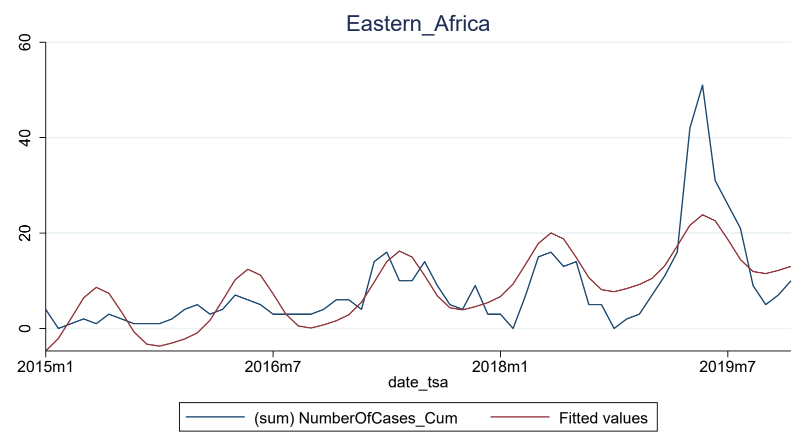

Number of travel-related cases of dengue among travellers arriving from **Western Africa** and rates of infection (TIR), by month, and 3-, 6- and 12-month moving averages, 2015-2019

Rate of infection

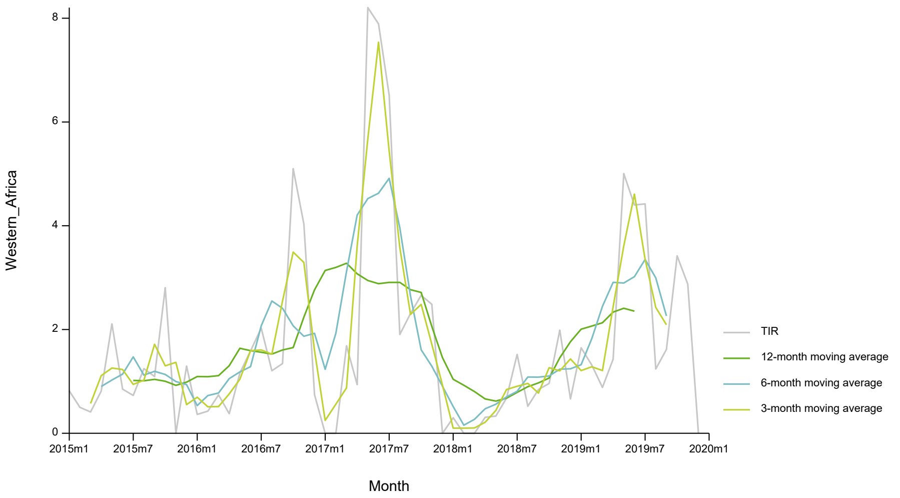

Number of cases

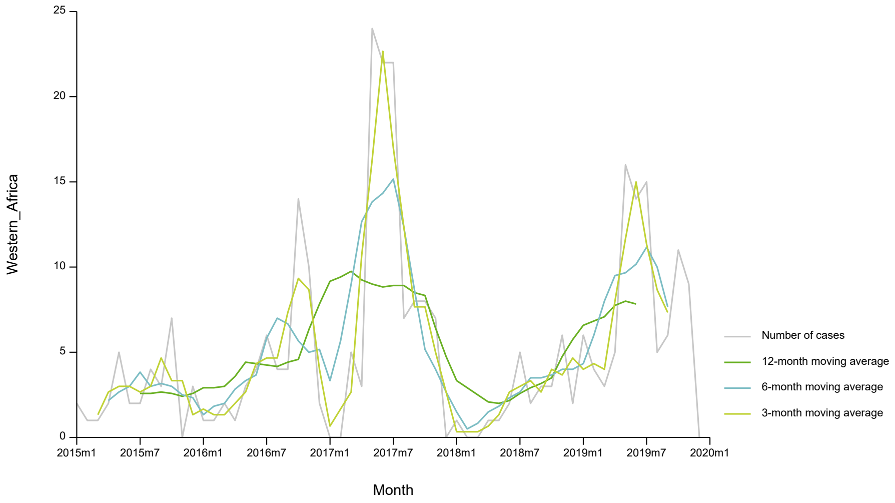

Trend adjusted for seasonality

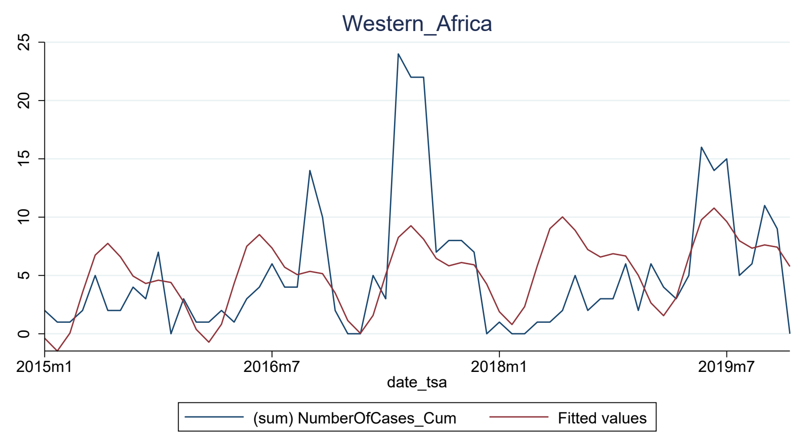

Number of travel-related cases of dengue among travellers arriving from **Polynesia** and rates of infection (TIR), by month, and 3-, 6- and 12-month moving averages, 2015-2019

Rate of infection

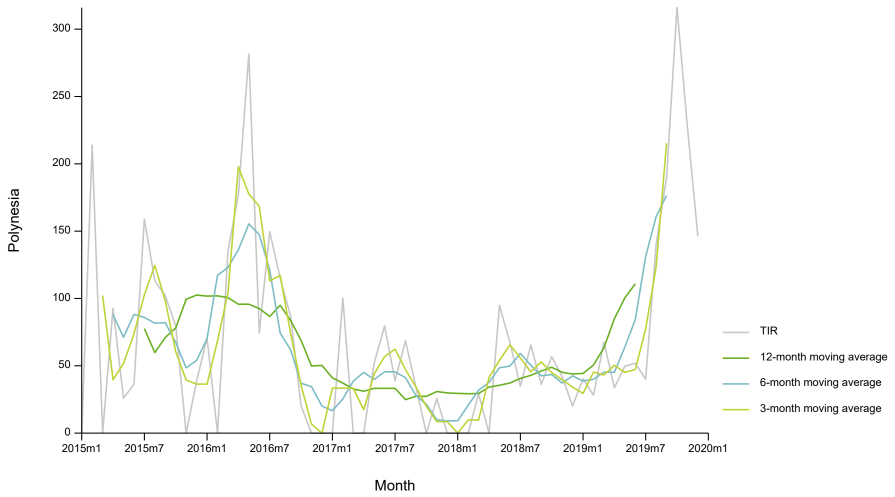

Number of cases

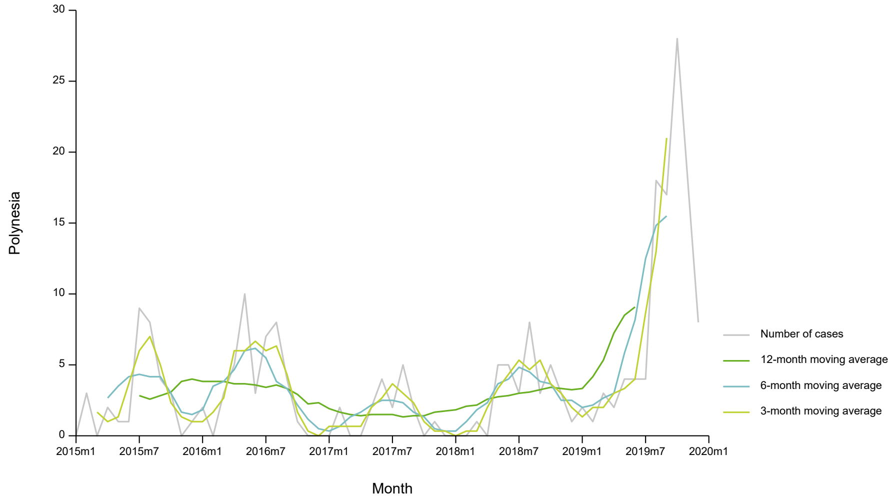

Trend adjusted for seasonality

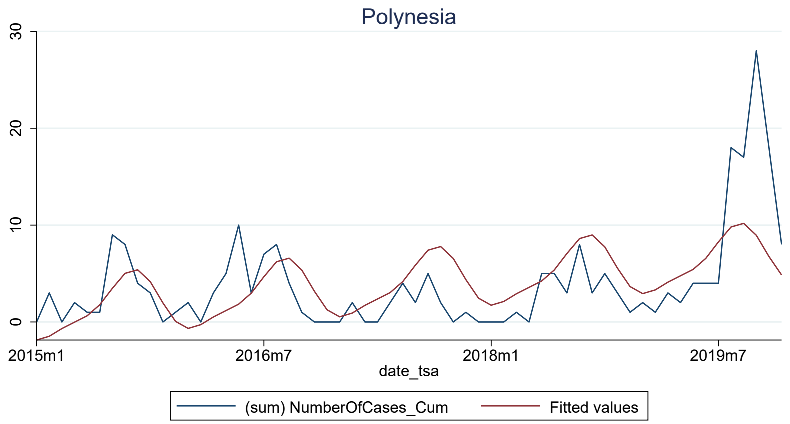

Regression coefficients, corresponding 95% confidence intervals obtained by the harmonic regression analysis using **the number of cases** by region

	Caribbean		Central_America		South_America		Eastern_Africa		Western_Africa		South_Eastern_Asia		Southern_Asia		Polynesia	
	**coeff.**	**95%CI**	**coeff.**	**95%CI**	**coeff.**	**95%CI**	**coeff.**	**95%CI**	**coeff.**	**95%CI**	**coeff.**	**95%CI**	**coeff.**	**95%CI**	**coeff.**	**95%CI**
**sin3**	0.006	(-6.803; 6.815)	-1.457	(-4.105; 1.191)	0.441	(-3.214; 4.096)	0.588	(-1.894; 3.071)	0.211	(-1.606; 2.027)	-5.091	(-18.99; 8.809)	-2.744	(-9.384; 3.895)	-0.577	(-2.235; 1.081)
**cos3**	-1.743	(-8.556; 5.070)	-1.010	(-3.659; 1.640)	-0.864	(-4.521; 2.793)	-0.953	(-3.437; 1.532)	-0.932	(-2.749; 0.886)	-4.715	(-18.62; 9.193)	-4.413	(-11.06; 2.230)	-0.834	(-2.493; 0.825)
**sin6**	-0.560	(-7.385; 6.265)	1.691	(-0.963; 4.345)	-0.062	(-3.726; 3.601)	-1.237	(-3.725; 1.251)	-1.273	(-3.094; 0.547)	13.130 *	(-0.800; 27.060)	-9.792 ***	(-16.45; -3.137)	-0.057	(-1.719; 1.606)
**cos6**	0.057	(-6.756; 6.870)	-2.243 *	(-4.892; 0.406)	-1.597	(-5.255; 2.060)	1.114	(-1.370; 3.598)	1.435	(-0.382; 3.253)	-14.180 **	(-28.09; -0.274)	-8.447 **	(-15.09: -1.804)	-0.867	(-2.527; 0.792)
**sin12**	-7.809 **	(-14.70 ; -0.921)	-6.231 ***	(-8.910; -3.552)	5.995 ***	(2.297; 9.693)	3.643 ***	(1.132; 6.155)	-1.931 **	(-3.769; -0.0936)	14.350 **	(0.288; 28.410)	-30.200 ***	(-36.920; -23.480)	-2.733 ***	(-4.411; -1.055)
**cos12**	5.765 *	(-1.048; 12.58)	0.071	(-2.579; 2.720)	-2.024	(-5.681; 1.634)	-5.733 ***	(-8.217; -3.249)	-2.694 ***	(-4.511; -0.876)	-17.710 **	(-31.610; -3.798)	9.364 ***	(2.721; 16.010)	-1.157	(-2.816; 0.502)
**trend**	0.460 ***	(0.177; 0.743)	0.076	(-0.033; 0.186)	-0.136 *	(-0.288; 0.0159)	0.319 ***	(0.216; 0.422)	0.065 *	(-0.0105; 0.140)	0.482 *	(-0.0954; 1.059)	0.547 ***	(0.271; 0.822)	0.101 ***	(0.0318; 0.169)
**Constant**	1.206	(-8.671; 11.08)	6.055 ***	(2.214; 9.895)	15.040 ***	(9.742; 20.35)	-1.451	(-5.053; 2.150)	3.187 **	(0.552; 5.822)	78.990 ***	(58.830; 99.150)	24.640 ***	(15.010; 34.270)	0.864	(-1.541; 3.270)
**Observations**	60		60		60		60		60		60		60		60	
**R-squared**	0.300		0.383		0.264		0.557		0.309		0.281		0.727		0.339	

*** p<0.01, ** p<0.05, * p<0.1

Regression coefficients, corresponding 95% confidence intervals obtained by the harmonic regression analysis using the **TIR**, by region

VARIABLES	Caribbean		Central_America		South_America		Eastern_Africa		Western_Africa		South_Eastern_Asia		Southern_Asia		Polynesia	
sin3	0.304	(-1.798; 2.406)	-0.499	(-1.629; 0.631)	0.060	(-0.762; 0.883)	0.107	(-1.030; 1.245)	0.025	(-0.585; 0.635)	-0.945	(-3.030; 1.141)	-0.294	(-1.529; 0.942)	-9.518	(-36.17; 17.14)
cos3	-0.341	(-2.445; 1.762)	-0.475	(-1.606; 0.656)	-0.177	(-1.000; 0.646)	-0.324	(-1.462; 0.814)	-0.273	(-0.884; 0.337)	-0.223	(-2.309; 1.864)	-0.754	(-1.990; 0.483)	-9.571	(-36.24; 17.10)
sin6	-0.885	(-2.991; 1.222)	0.386	(-0.747; 1.519)	-0.079	(-0.904; 0.745)	-1.066 *	(-2.206; 0.0737)	-0.564 *	(-1.176; 0.0480)	1.001	(-1.089; 3.091)	-2.159 ***	(-3.398; -0.921)	-5.332	(-32.05; 21.38)
cos6	-0.216	(-2.319; 1.887)	-0.856	(-1.987; 0.275)	-0.446	(-1.269; 0.377)	0.693	(-0.445; 1.831)	0.549 *	(-0.0615; 1.160)	-0.843	(-2.929; 1.243)	-1.112 *	(-2.348; 0.125)	-10.550	(-37.22; 16.12)
sin12	-2.921 ***	(-5.047; -0.795)	-2.698 ***	(-3.841; -1.554)	1.476 ***	(0.644; 2.308)	1.995 ***	(0.845; 3.146)	-0.611 *	(-1.228; 0.00654)	1.115	(-0.995; 3.224)	-6.063 ***	(-7.313; -4.813)	-17.700	(-44.66; 9.266)
cos12	1.701	(-0.402; 3.804)	0.474	(-0.657; 1.605)	-0.299	(-1.122; 0.524)	-2.706 ***	(-3.844; -1.568)	-0.822 ***	(-1.433; -0.211)	-3.931 ***	(-6.018; -1.845)	1.785 ***	(0.549; 3.021)	-18.560	(-45.23; 8.105)
trend	0.112 **	(0.0248; 0.199)	-0.007	(-0.0543; 0.0396)	-0.049 ***	(-0.0830; -0.0147)	0.124 ***	(0.0765; 0.171)	0.013	(-0.0125; 0.0382)	-0.020	(-0.106; 0.0670)	0.062 **	(0.0104; 0.113)	0.200	(-0.906; 1.307)
Constant	1.201	(-1.848; 4.249)	4.117 ***	(2.478; 5.756)	3.802 ***	(2.609; 4.995)	-0.094	(-1.744; 1.556)	1.320 ***	(0.435; 2.206)	16.410 ***	(13.38; 19.43)	6.213 ***	(4.421; 8.006)	63.020 ***	(24.36; 101.7)
Observations	60	60	60	60	60	60	60	60	60	60	60	60	60	60	60	60
R-squared	0.287	0.287	0.347	0.347	0.343	0.343	0.548	0.548	0.285	0.285	0.263	0.263	0.725	0.725	0.1	0.1

Number of travel-related cases of dengue among travellers arriving from **Eastern Asia** and rates of infection (TIR), by month, and 3-, 6- and 12-month moving averages, 2015-2019

Rate of infection

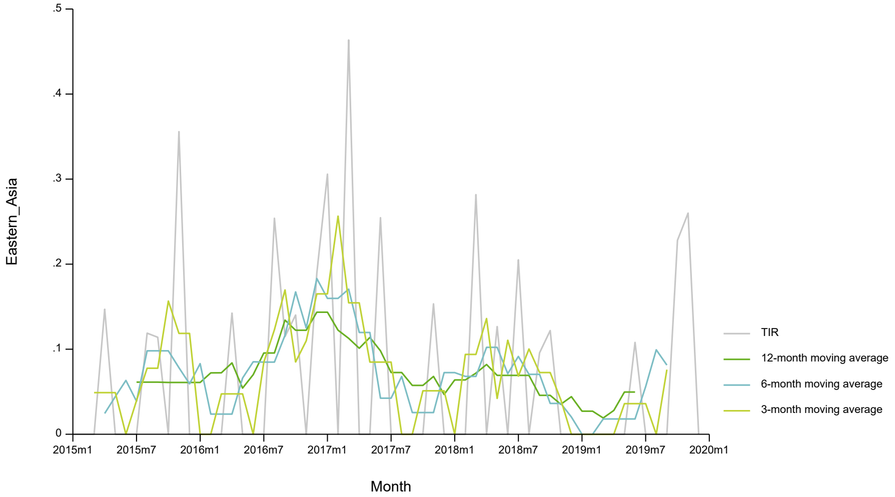

Number of cases

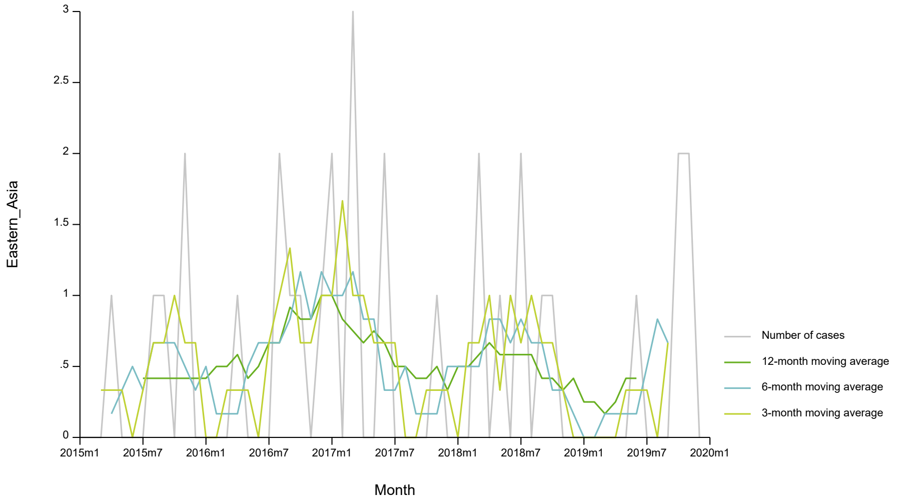

Trend adjusted for seasonality

Not applicable

<10 cases/per month

Number of travel-related cases of dengue among travellers arriving from **Melanesia** and rates of infection (TIR), by month, and 3-, 6- and 12-month moving averages, 2015-2019

Rate of infection

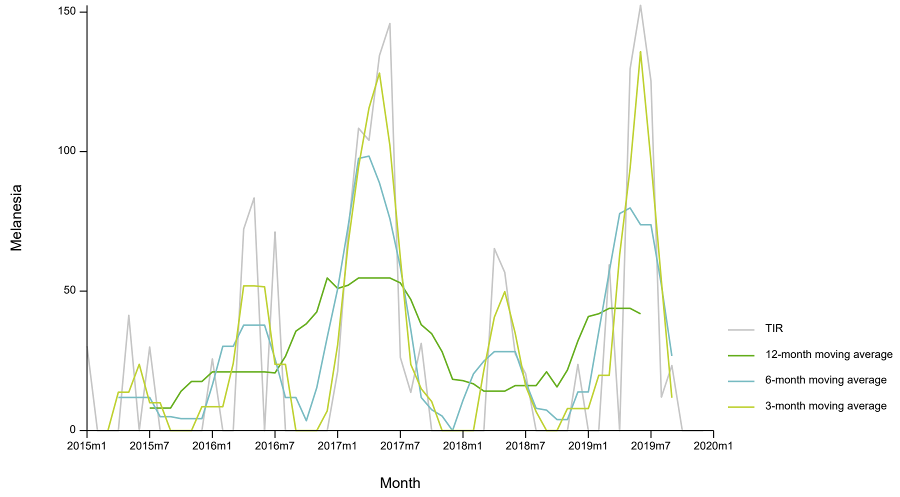

Number of cases

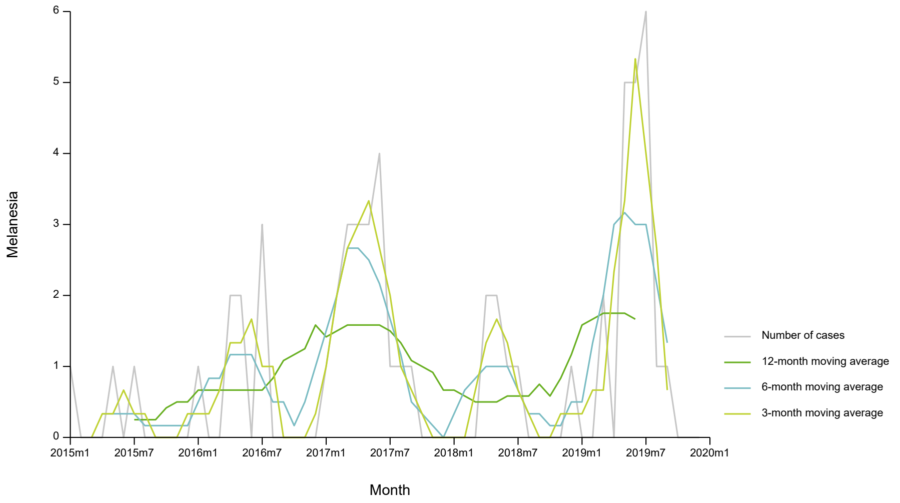

Trend adjusted for seasonality

Not applicable

<10 cases/per month

Number of travel-related cases of dengue among travellers arriving from **Micronesia** and rates of infection (TIR), by month, and 3-, 6- and 12-month moving averages, 2015-2019

Rate of infection

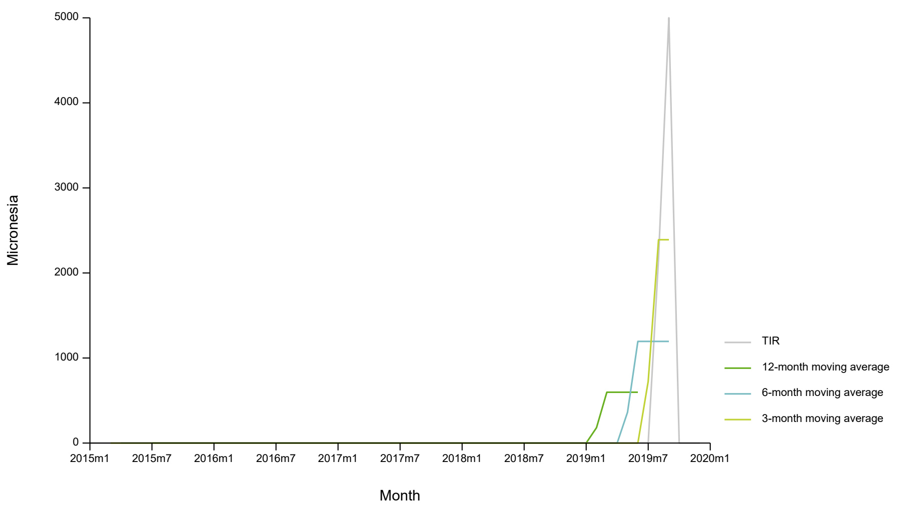

Number of cases

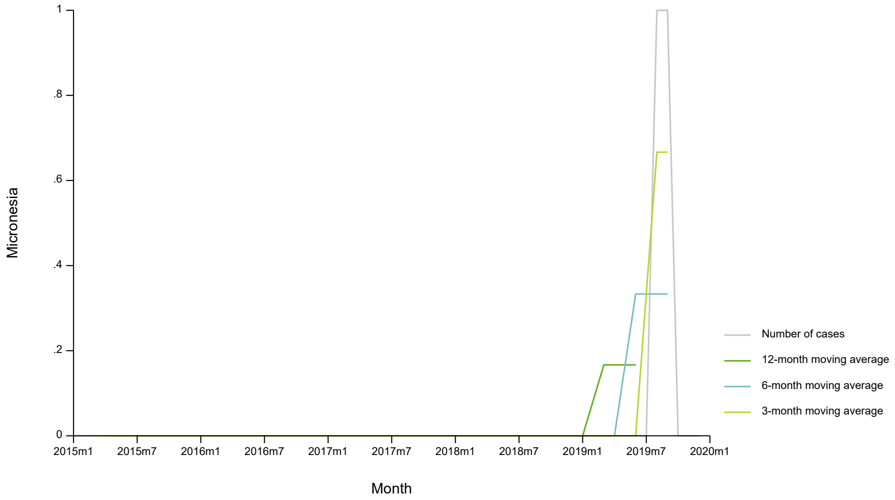

Trend adjusted for seasonality

Not applicable

<10 cases/per month

Number of travel-related cases of dengue among travellers arriving from **Middle Africa** and rates of infection (TIR), by month, and 3-, 6- and 12-month moving averages, 2015-2019

Rate of infection

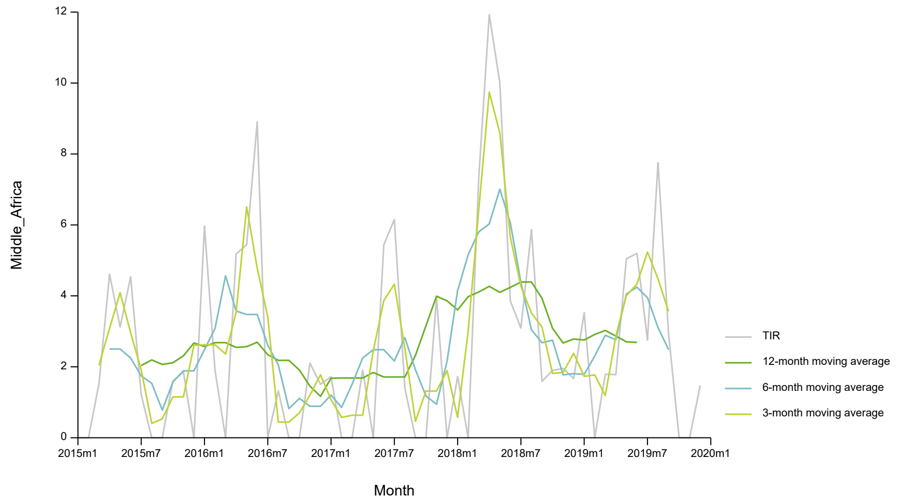

Number of cases

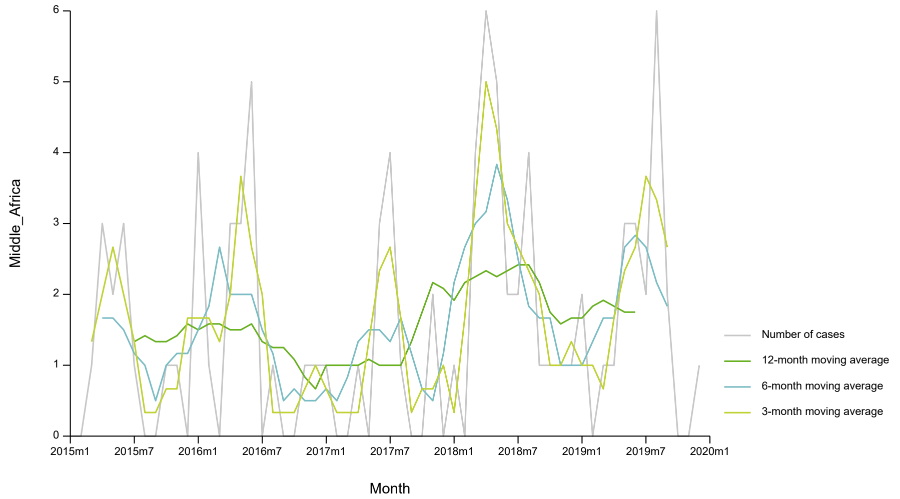

Trend adjusted for seasonality

Not applicable

<10 cases/per month

Number of travel-related cases of dengue among travellers arriving from **Northern Africa** and rates of infection (TIR), by month, and 3-, 6- and 12-month moving averages, 2015-2019

Rate of infection

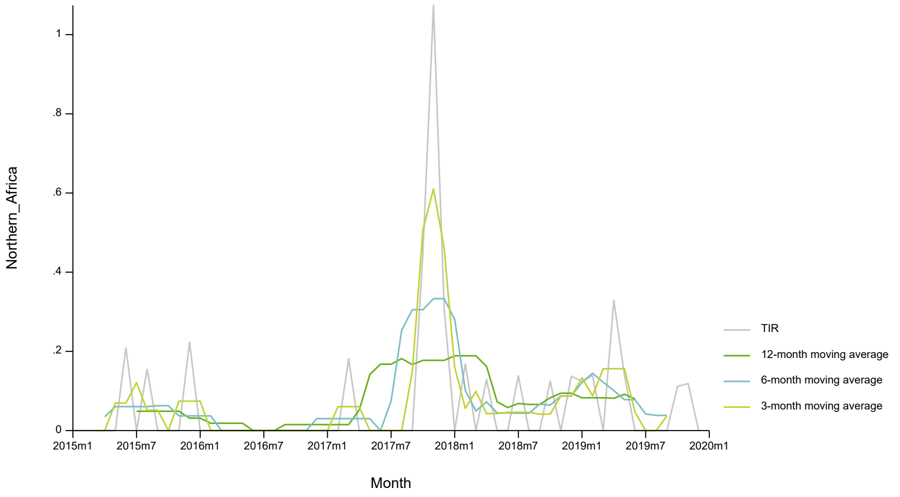

Number of cases

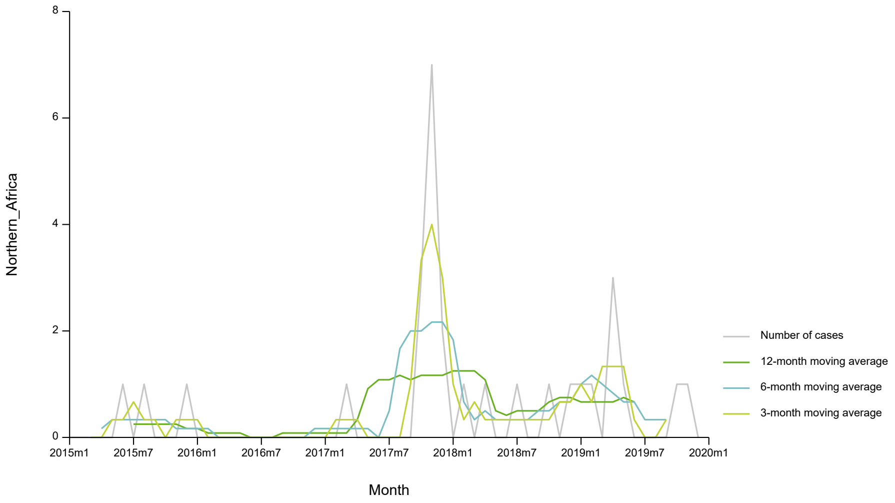

Trend adjusted for seasonality

Not applicable

<10 cases/per month

Number of travel-related cases of dengue among travellers arriving from **Northern America** and rates of infection (TIR), by month, and 3-, 6- and 12-month moving averages, 2015-2019

Rate of infection

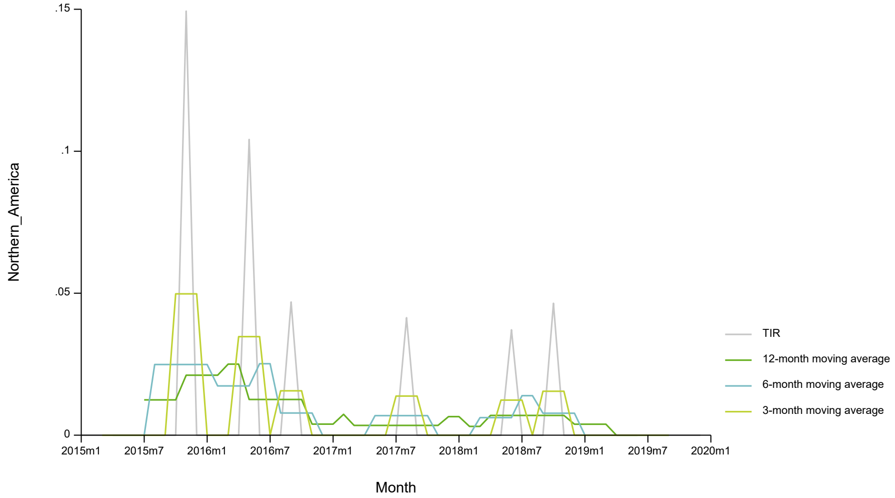

Number of cases

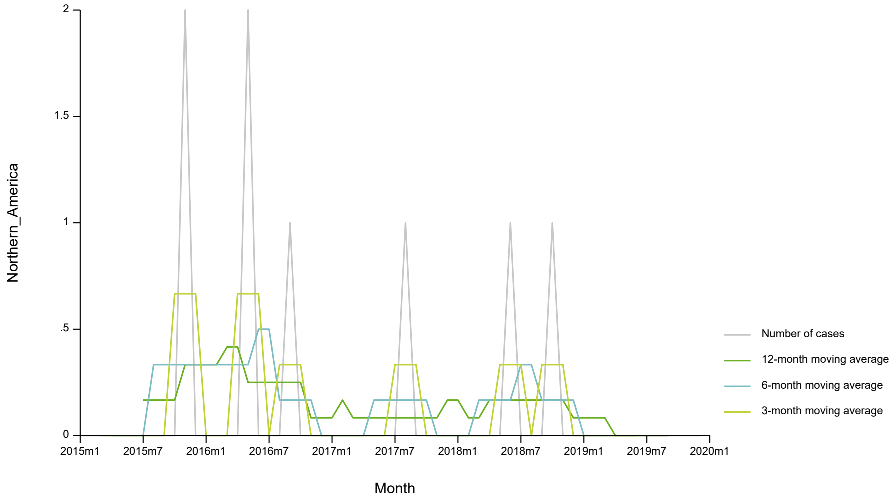

Trend adjusted for seasonality

Not applicable

<10 cases/per month

Number of travel-related cases of dengue among travellers arriving from **Southern Africa** and rates of infection (TIR), by month, and 3-, 6- and 12-month moving averages, 2015-2019

Rate of infection

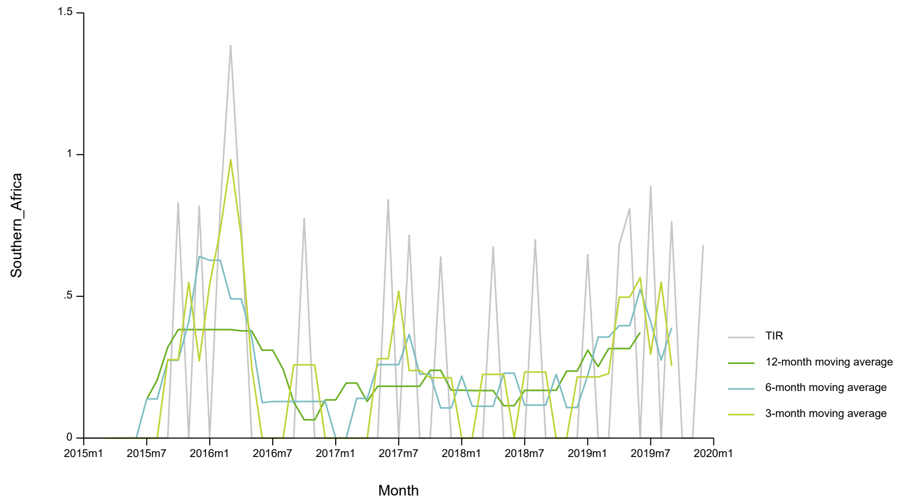

Number of cases

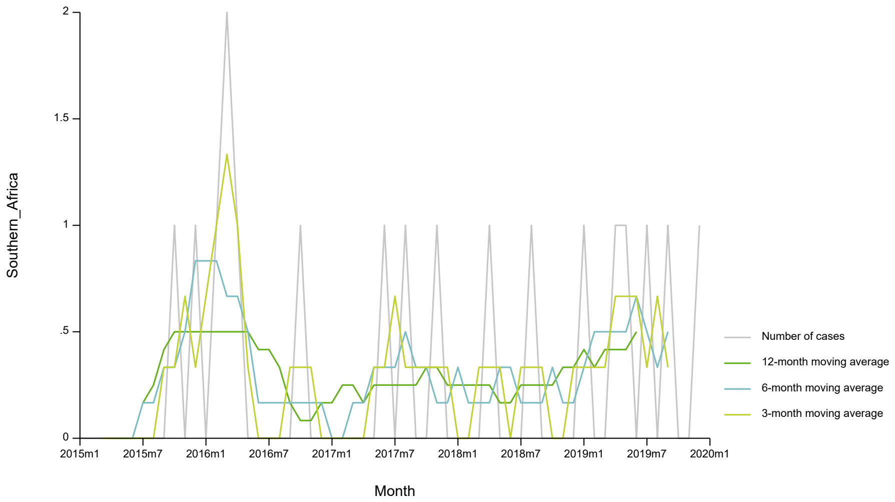

Trend adjusted for seasonality

Not applicable

<10 cases/per month

Number of travel-related cases of dengue among travellers arriving from **Western Asia** and rates of infection (TIR), by month, and 3-, 6- and 12-month moving averages, 2015-2019

Rate of infection

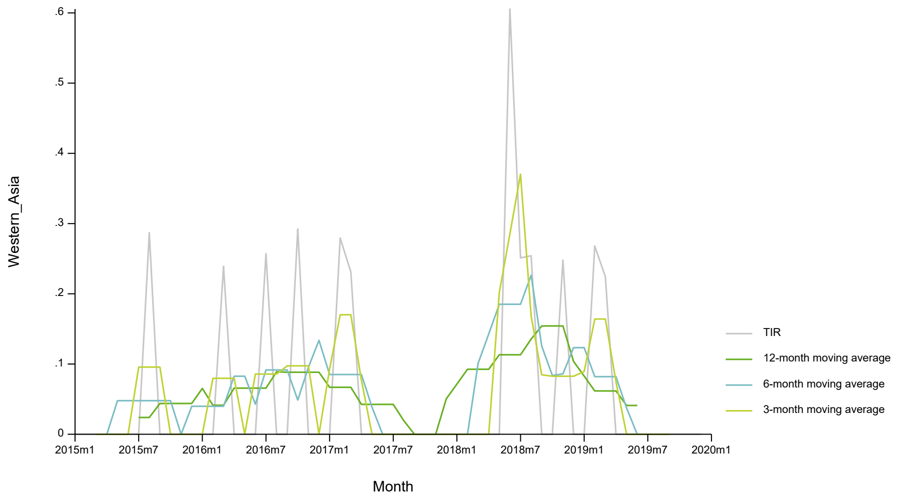

Number of cases

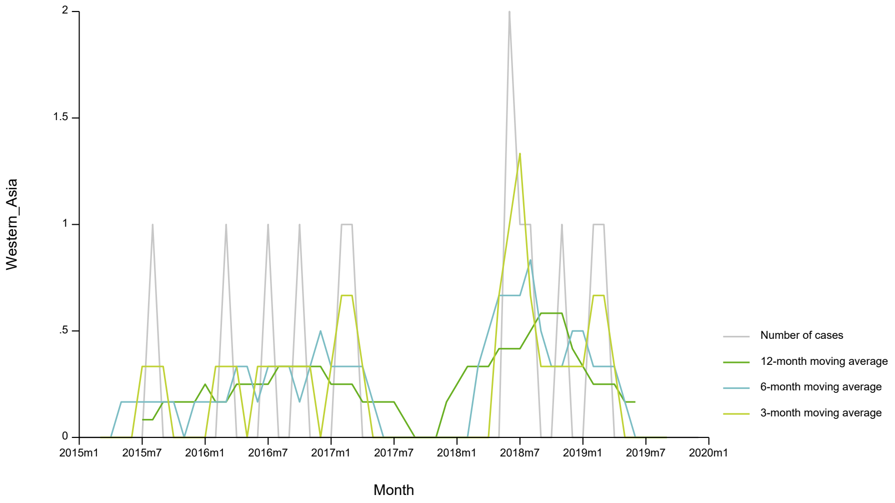

Trend adjusted for seasonality

Not applicable

<10 cases/per month

Number of travel-related cases of dengue among travellers arriving from **Australia and New Zealand** and rates of infection (TIR), by month, and 3-, 6- and 12-month moving averages, 2015-2019

Rate of infection

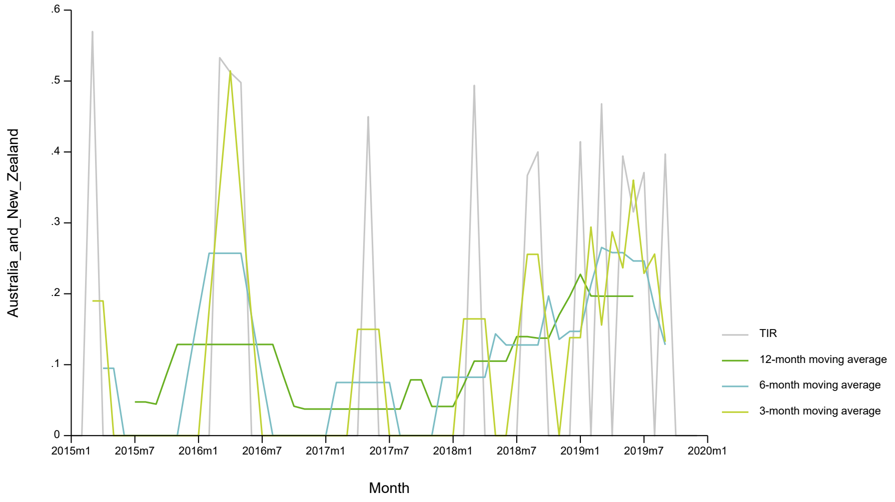

Number of cases

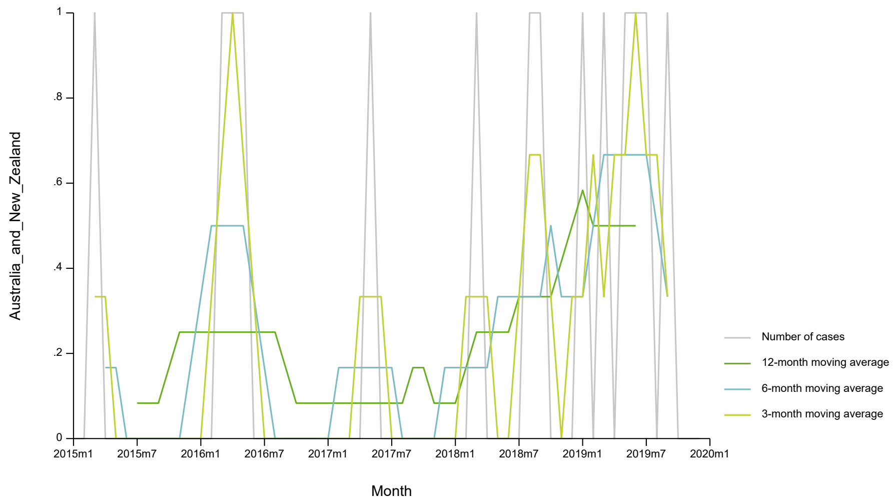

Trend adjusted for seasonality

Not applicable

<10 cases/per month

Part C – Linear regression analysis

Association between disease incidence rate and rates of infections among travellers, by selected countries, 2015-2019

Country	Coefficient of regression	Intercept	Confidence interval lower 95%	Confidence interval upper 95%	p value	R square
Cuba	1.44	-15.09	0.51	2.37	0.02	0.89
French Polynesia	0.11	17.27	0.06	0.16	0.01	0.94
Réunion	0.01	-0.08	0.007	0.01	<0.01	0.99
Thailand	0.01	17.81	-0.14	0.16	0.80	0.02

Rates of infection among travellers and incidence rates among the local population in Cuba, French Polynesia, Réunion and Thailand, per country and per year, 2015-2019

Country	Year	Rate of infection among travellers	Incidence rate among the local population
Cuba	2015	10.8	14.9
Cuba	2016	5.8	16.2
Cuba	2017	1.4	11.0
Cuba	2018	7.7	18.8
Cuba	2019	27.9	28.8
French Polynesia	2015	69.0	385.2
French Polynesia	2016	94.7	762.3
French Polynesia	2017	36.1	296.6
French Polynesia	2018	45.4	230.5
French Polynesia	2019	121.7	975.7
Réunion	2015	0.0	1.4
Réunion	2016	0.2	25.7
Réunion	2017	0.7	11.4
Réunion	2018	5.2	785.1
Réunion	2019	17.9	2110.1
Thailand	2015	17.8	210.9
Thailand	2016	19.3	92.7
Thailand	2017	14.6	76.9
Thailand	2018	17.9	79.3
Thailand	2019	26.9	124.1
